# Toward ultracentrifugation-free processing and long-term preservation of outer membrane vesicles by culture optimization, tangential flow filtration, diafiltration, and lyophilization

**DOI:** 10.3389/fmicb.2026.1927950

**Published:** 2026-08-31

**Authors:** Andres Silva, Ignacio Fuentes, Jessenia C. Melio, Francisco Parra, Diana Majluf, Diego Rojas, Felipe M. Llancalahuen, Maria J. Barros, Katina Schinnerling, Fernando Gil, Iván L. Calderón, Juan A. Fuentes

**Affiliations:** 1 Laboratorio de Genética y Patogénesis Bacteriana, Centro de Investigación de Resiliencia a Pandemias, Facultad de Ciencias de la Vida, Universidad Andrés Bello, Santiago, Chile; 2 Doctorado en Biotecnología, Facultad de Ciencias de la Vida, Universidad Andrés Bello, Santiago, Chile; 3 Doctorado en Biociencias Moleculares, Facultad de Ciencias de la Vida, Universidad Andrés Bello, Santiago, Chile; 4 Magíster en Biotecnología y Ciencias de la Vida, Facultad de Ciencias de la Vida, Universidad Andrés Bello, Santiago, Chile; 5 Translational Research Laboratory in Intensive Care Medicine, Departamento de Medicina Intensiva, Facultad de Medicina, Pontificia Universidad Católica de Chile, Santiago, Chile; 6 Laboratorio de RNAs Bacterianos, Centro de Investigación de Resiliencia a Pandemias, Facultad de Ciencias de la Vida, Universidad Andrés Bello, Santiago, Chile; 7 Laboratorio de Inmunología Traslacional, Centro de Investigación de Resiliencia a Pandemias, Facultad de Ciencias de la Vida e Instituto de Salud Pública, Universidad Andrés Bello, Santiago, Chile; 8 School of Medicine, Faculty of Medicine, Universidad de los Andes, Santiago, Chile; 9 Microbiota-Host Interactions and Clostridia Research Group, Center for Biomedical Research and Innovation (CIIB), Universidad de los Andes, Santiago, Chile

**Keywords:** bacterial vesicles, diafiltration, flagellin, lyophilization, microbial biotechnology, OMV enrichment, outer membrane vesicles (OMVs), *Salmonella* Typhi

## Abstract

Outer membrane vesicles (OMVs) are bacterial membrane nanoparticles with applications in host–microbe interaction studies, vaccines, molecular delivery, and microbial biotechnology. However, OMV workflows often rely on ultracentrifugation, limiting accessibility and process integration. Here, we evaluated a laboratory-scale workflow for *Salmonella enterica* serovar Typhi that sequentially combined culture optimization, TFF-based preconcentration, diafiltration performed in the same TFF system, and lyophilization. We first assessed how culture volume and agitation affected apparent OMV-associated recovery, flagellin carryover, vesicle morphology, and epithelial cell-associated fluorescence. Higher agitation increased protein- and particle-associated recovery readouts but also promoted co-isolation of flagellin-containing filaments, whereas low agitation yielded morphologically better-defined preparations with reduced flagellin carryover and higher cell-associated and trypan blue-resistant fluorescence. TFF and normal-flow filtration were then compared as preconcentration steps before ultracentrifugation and were comparable across morphology, particle size, global protein profiles, and recovery readouts, supporting selection of TFF for workflow integration. During refrigerated, frozen, and lyophilized storage, bulk protein- and lipid-associated signals remained detectable, whereas size distribution, clustering, and surface charge changed over time. Lyophilized preparations retained detectable β-lactamase activity, mCherry fluorescence, and epithelial cell-associated fluorescence after reconstitution. Finally, an integrated ultracentrifugation-free workflow, in which TFF preconcentration was followed by diafiltration in the same TFF system and subsequent lyophilization, preserved multiple OMV-associated structural and functional readouts without ultracentrifugation, including recognizable vesicular morphology, nanoscale particle size, global protein profile, membrane-protected mCherry-FLAG cargo, and detectable fluorescent reporter signal. Compared with ultracentrifugation-based controls, the integrated workflow showed a lower total-protein readout, whereas lipid- and particle-associated readouts were not significantly reduced. These findings define a sequential, ultracentrifugation-free processing framework for research-grade *S.* Typhi OMVs and support further development of scale-compatible workflows while identifying colloidal stability, cargo-specific retention, and compositional definition as priorities for subsequent validation.

## 1 Introduction

Outer membrane vesicles (OMVs) are a major class of bacterial extracellular vesicles naturally released by Gram-negative bacteria through outward blebbing of the outer membrane (Sartorio et al., 2021; Juodeikis and Carding, 2022). By carrying outer membrane and periplasmic components, including lipopolysaccharide, phospholipids, outer membrane proteins, periplasmic enzymes, peptidoglycan fragments, and nucleic acids, OMVs preserve key structural and functional features of the parental bacterial envelope while providing a protected cargo-delivery vehicle enriched in immunostimulatory envelope-associated molecules (Schwechheimer and Kuehn, 2015; Sartorio et al., 2021; Juodeikis and Carding, 2022). These properties have positioned OMVs as versatile platforms for microbiological research, vaccine development, antigen and protein delivery, and microbial biotechnology (Schwechheimer and Kuehn, 2015; Gerritzen et al., 2017; Sartorio et al., 2021). However, their broader experimental, biotechnological, and translational use depends on the ability to generate preparations that are not only abundant, but also reproducible, sufficiently defined, and compatible with scalable processing and storage workflows (Klimentová and Stulík, 2015; Schwechheimer and Kuehn, 2015; Gerritzen et al., 2017).

A major bottleneck in OMV research is that isolation workflows have traditionally been optimized for analytical recovery rather than for controlled product quality. Conventional OMV isolation typically relies on clarification, membrane filtration, concentration, ultracentrifugation-based pelleting, and resuspension; although widely used, these procedures are labor-intensive, equipment-dependent, poorly suited for scale-compatible workflows, and sensitive to operator- and process-dependent variation (Klimentová and Stulík, 2015; Choi and Lee, 2025). More importantly, pelleting-based workflows may co-recover soluble proteins, protein aggregates, flagellar fragments, membrane debris, and other non-vesicular material, thereby inflating apparent OMV yield and confounding biochemical, physicochemical, and functional readouts (Klimentová and Stulík, 2015; Reimer et al., 2021; Choi and Lee, 2025). Accordingly, OMV processing should not be framed solely as a vesicle-recovery problem, because apparent recovery and preparation purity must be evaluated together with vesicle morphology, contaminant carryover, particle characteristics, cargo-associated functionality, and recipient-cell interaction. Importantly, these quality attributes are not determined only during isolation, because culture conditions can modify both the OMV-associated material and the impurity profile entering the downstream purification workflow. Therefore, conditions that enhance apparent vesicle recovery may also alter the impurity profile by increasing the abundance of co-isolated flagellar structures, soluble proteins, membrane debris, or other non-vesicular material. Bacterial physiology and environmental conditions influence vesicle release, size, cargo composition, surface properties, and interactions with recipient cells (Orench-Rivera and Kuehn, 2016). Growth phase has been shown to alter membrane-vesicle physicochemical properties and cell-association in *Pseudomonas aeruginosa*, as well as OMV size, protein composition, and cargo packaging in *Helicobacter pylori* (Tashiro et al., 2010; Zavan et al., 2019), whereas dissolved oxygen tension can markedly increase spontaneous OMV productivity in *Neisseria meningitidis* (Gerritzen et al., 2018). In shake-flask cultures, agitation speed and filling volume influence mixing, oxygen transfer, gas exchange, and potentially shear-associated release of non-vesicular material (Takahashi and Aoyagi, 2020). Therefore, increasing culture output cannot be assumed to result in more favorable OMV quality attributes: conditions that enhance apparent vesicle recovery may also increase co-isolated contaminants, alter vesicle heterogeneity, or compromise downstream functional performance.

Flagellin carryover is particularly relevant for OMVs derived from flagellated bacteria such as *Salmonella*, where flagella and flagellin can co-purify with vesicle preparations and compromise their biochemical and functional interpretation. In *Salmonella enterica* serovar Typhimurium, flagellar material has been reported to remain in OMV pellets after purification, and flagellin-deficient strains have been used to improve antigenic definition and reduce flagellin-driven immune responses in OMV-based vaccine formulations (Liu et al., 2016a; Liu et al., 2018). Although flagellin carryover has been most clearly documented in *S.* Typhimurium, it is also potentially relevant to flagellated *Salmonella* backgrounds such as *S.* Typhi, where flagellin carryover may similarly compromise OMV product definition. Because flagellin is a potent agonist of Toll-like receptor 5-dependent innate immune signaling, its co-isolation may dominate or confound host-cell responses otherwise attributed to OMVs (Hayashi et al., 2001; Skountzou et al., 2010). Although genetic deletion of flagellin can reduce flagellin-associated contaminants and improve the compositional specificity of OMV preparations, it also alters the genotype and surface structures of the producing strain, potentially affecting vesicle composition and host-cell responses. Therefore, upstream process control represents a complementary strategy to reduce flagellin carryover while retaining the wild-type genetic background of the producing bacterium.

Downstream processing adds a further layer of complexity to OMV quality control. Tangential flow filtration (TFF) is a scale-compatible membrane-based unit operation in which fluid flows parallel to the membrane surface, reducing fouling relative to dead-end filtration and enabling both concentration and solution exchange through diafiltration (Busatto et al., 2018; Visan et al., 2022). Beyond concentration and solution exchange, preservation introduces an additional quality-control challenge. Storage can alter vesicle concentration, size distribution, aggregation state, surface charge, cargo integrity, and biological activity, and these attributes may change independently (Jeyaram and Jay, 2017; Gelibter et al., 2022). Lyophilization is therefore attractive because it may reduce the need for refrigerated or frozen storage during storage, transportation, and distribution; however, dehydration and reconstitution can impose physical stress on vesicles, making formulation and lyoprotectant selection essential (Trenkenschuh et al., 2022; Ahmadian et al., 2024). Evidence from extracellular vesicle systems indicates that sugars such as sucrose or trehalose can improve colloidal stability after freeze-drying, while enzyme-loaded bacterial OMVs can preserve cargo activity after lyophilization (Alves et al., 2016; Trenkenschuh et al., 2022; Ahmadian et al., 2024).

Although TFF has been used for both preconcentration and diafiltration, and lyophilization has also been explored in extracellular-vesicle processing, their sequential integration into a single ultracentrifugation-free OMV workflow has, to our knowledge, not been systematically evaluated (Watson et al., 2021; Visan et al., 2022; Torres-Vanegas et al., 2024). It therefore remains unknown whether such an integrated workflow retains key structural and cargo-associated attributes together with measurable cell-interaction properties.

*Salmonella enterica* serovar Typhi is a human-restricted pathogen and the etiological agent of typhoid fever. *S.* Typhi-derived OMVs have been explored as vaccine-relevant and engineered delivery-oriented platforms (Howlader et al., 2018; Haque et al., 2021; Fuentes et al., 2025). In addition, *S.* Typhi provides a relevant native OMV-producing background in which vesicle recovery may be more modest than in hypervesiculating derivatives, while flagellin carryover may complicate biochemical and functional interpretation. Previous work has identified genetic determinants of OMV biogenesis in *S.* Typhi and shown that hypervesiculating mutants can increase OMV yield and cargo delivery capacity, although these genetic modifications may also alter vesicle composition, population heterogeneity, and recipient-cell interactions (Nevermann et al., 2019; Marchant et al., 2021; Fuentes et al., 2025).

In this study, we used *Salmonella enterica* serovar Typhi as a native OMV-producing Gram-negative model to evaluate an integrated OMV-processing workflow. We asked whether upstream culture-condition control, TFF-based preconcentration, diafiltration performed in the same TFF system, and lyophilization could be progressively integrated into an ultracentrifugation-free workflow. The study was organized sequentially to link each process variable with a defined set of process-performance readouts and OMV quality attributes. We first varied agitation speed and culture volume and examined their effects on bacterial growth, apparent preparation recovery based on protein-, lipid-, and particle-associated measurements, flagellin co-isolation, vesicle morphology, and epithelial cell-associated fluorescence, including its trypan blue-resistant fraction. We then compared normal-flow filtration and TFF as preconcentration strategies before ultracentrifugation, using morphology, particle size, global protein profile, and protein-, lipid-, and particle-associated recovery as comparative readouts. Next, we assessed how refrigerated, frozen, and lyophilized storage affected structural, physicochemical, cargo-associated, and cell-interaction properties. Finally, TFF preconcentration, followed by diafiltration in the same TFF system, and subsequent lyophilization, was integrated into an ultracentrifugation-free workflow and evaluated for apparent recovery, morphology, particle size, protein profile, cargo protection, and detectable reporter functionality.

## 2 Materials and methods

### 2.1 Overall experimental design

**FIGURE 1:**
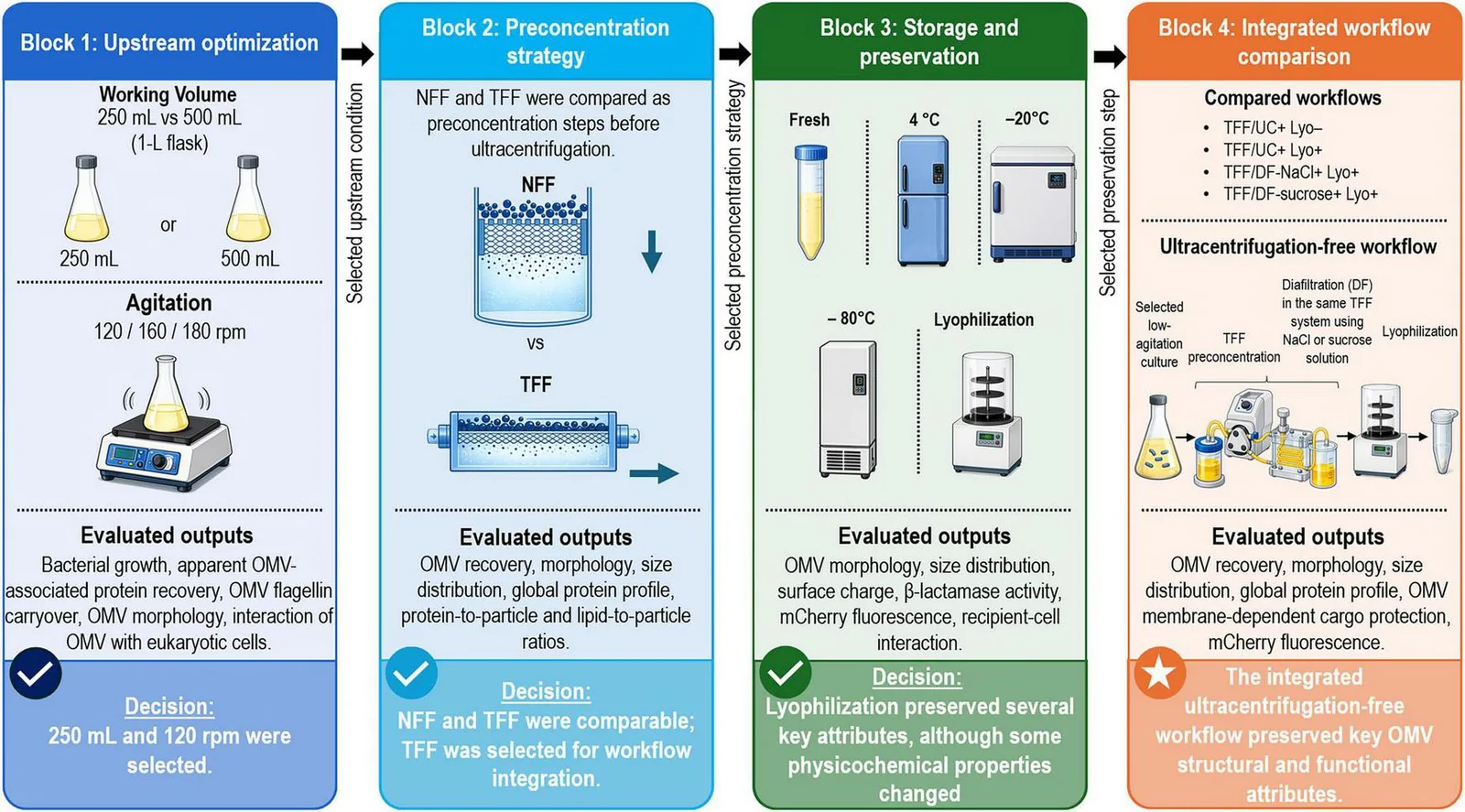
Sequential experimental design leading to the integrated ultracentrifugation-free OMV workflow. The study was organized into four sequential experimental blocks: upstream culture optimization, comparison of preconcentration strategies, evaluation of storage and preservation conditions, and integration of the final ultracentrifugation-free workflow. The outcome of each block informed the experimental conditions used in the subsequent stage. The process variables, conditions compared, decision criteria, and corresponding process-performance readouts and OMV quality attributes are summarized in the figure and detailed in Table 1.

The study followed a sequential process-development strategy in which upstream production, downstream concentration, preservation, and final workflow integration were evaluated as interconnected stages of OMV processing. Apparent protein-, lipid-, and particle-associated recovery was used to assess process performance, whereas morphology, flagellin carryover, particle-size distribution, surface charge, cargo-associated functionality, and recipient-cell interaction were used to assess complementary attributes of the final OMV preparations. The sequential experimental design and the decision points linking the four experimental blocks are summarized in Figure 1, while the process variables, conditions compared, and corresponding readouts are detailed in Table 1.

**TABLE 1:** Sequential experimental design linking process variables to process-performance readouts and OMV quality attributes.

Experimental block	Process parameter varied	Conditions compared	Process-performance readouts and OMV quality attributes assessed
1. Upstream culture optimization	Flask filling volume and agitation speed	Growth kinetics were evaluated using 250- or 500-mL cultures at 120 or 180 rpm. Protein- and lipid-associated recovery was assessed in 250-mL cultures at 120, 160, or 180 rpm, whereas particle-associated recovery and epithelial cell-associated fluorescence were compared at 120 and 180 rpm. Vesicle morphology was examined across 250- and 500-mL cultures at 120, 160, or 180 rpm.	Process performance: bacterial growth; protein-, lipid-, and particle-associated recovery. OMV quality attributes: vesicle morphology; flagellin carryover; epithelial cell-associated and trypan blue-resistant fluorescence.
2. Preconcentration strategy	Filtration configuration	TFF versus NFF; both followed by ultracentrifugation	Process performance: particle concentration; protein-, lipid-, and particle-associated recovery. OMV quality attributes: vesicle morphology; particle-size distribution; global protein profile; protein-to-particle and lipid-to-particle ratios.
3. Storage and preservation	Storage condition and duration	4 °C, −20 °C, −80 °C, or lyophilized; Day 0 to Day 120	Process performance: protein- and lipid-associated signal retention. OMV quality attributes: vesicle morphology; TEM-derived size distribution; zeta potential; β-lactamase activity; mCherry fluorescence; epithelial cell-associated and trypan blue-resistant fluorescence.
4. Integrated process comparison	Downstream unit-operation sequence, diafiltration solution, and lyophilization status	TFF/UC+Lyo−; TFF/UC+Lyo+; TFF/DF-NaCl+Lyo+; TFF/DF-sucrose+Lyo+	Process performance: protein-, lipid-, and particle-associated recovery; particle concentration. OMV quality attributes: vesicle morphology; particle-size distribution; global protein profile; membrane-dependent cargo protection; mCherry fluorescence.

TFF, tangential flow filtration; NFF, normal-flow filtration; UC, ultracentrifugation; DF, diafiltration; Lyo, lyophilization. “+” and “−” indicate inclusion or omission of the indicated unit operation, respectively.

### 2.2 Bacterial strains and culture conditions

*Salmonella enterica* subsp. enterica serovar Typhi strain STH2370 (*S*. Typhi WT) (Valenzuela et al., 2014; Nevermann et al., 2019; Marchant et al., 2021; Marchant et al., 2024; Fuentes et al., 2025) was obtained from the Infectious Diseases Hospital Lucio Cordova, Chile. The *S*. Typhi *att*_*BG1*_::pNFB9 mutant and *S*. Typhi/pBSP*_*ompA*_-mcherry* have been previously described (Nevermann et al., 2019; Fuentes et al., 2025). *S*. Typhi Δ*fliC*::FRT strain was constructed using Red-Swap recombination (Datsenko and Wanner, 2000). Primers H1P1 fliC and H2P2 fliC were used for amplification of the *aph* resistance cassette and construction of the Δ*fliC* mutant by Red-Swap recombination, followed by cassette excision to generate the Δ*fliC*::FRT strain. Primers F fliC and R fliC were used to verify the mutation by PCR. Genotypic and phenotypic validation of the Δ*fliC*::FRT mutant are provided in the Supplementary Figure 1. All primer sequences used in this study are listed in Table 2.

**TABLE 2:** Primers used for construction and verification of the *S*. Typhi Δ*fliC*::FRT mutant.

Primer	Sequence
H1P1 fliC	ATGGCACAAGTCATTAATACAAACAGCCTGT CGCTGTTGACCTCCTTAGTTCCTATTCC
H2P2 fliC	CTGCATAGCCACCATCAATAACCTTACCGTT TTTATCCGATTGTGTAGGCTGGAGCTG
F fliC	GATCGGGGGAAGTGAAAAAT
R fliC	CGCTGCCTTGATTGTGTACC

Bacterial strains were routinely cultured in Luria–Bertani (LB) medium, composed of Bacto tryptone (10 g/L, Gibco, Thermo Fisher Scientific, Waltham, MA, United States), Bacto yeast extract (5 g/L, Gibco, Thermo Fisher Scientific, Waltham, MA, United States), and NaCl (5 g/L, Winkler, Lampa, Chile) in distilled water, and incubated at 37 °C with shaking in 1 L Erlenmeyer flasks. To ensure comparable starting material across downstream workflow comparisons, a standardized baseline culture volume of 1 L was used for all downstream OMV preparations, obtained by pooling four 250 mL cultures grown in parallel in 1 L Erlenmeyer flasks (120 rpm, 14 h, OD_600_ ≈ 1.0). In upstream optimization experiments, flask filling volumes (250 mL vs. 500 mL) and agitation speeds (120, 160, and 180 rpm) were evaluated to assess the impact of oxygen transfer, mixing, and shear stress on cell-culture productivity and OMV quality attributes. When necessary, the medium was supplemented with ampicillin (50 μg/mL, AppliChem GmbH, Darmstadt, Germany), gentamicin (12 μg/mL, Merck, Darmstadt, Germany), or X-gal (5-bromo-4-chloro-3-indolyl-β-D-galactopyranoside, 40 μg/mL, Merck, Darmstadt, Germany). Agar (Thermo Fisher Scientific, Waltham, MA, United States) was added at a final concentration of 15 g/L for solid media. To induce protein expression from the P*_*araBAD*_* promoter, the cultures were supplemented with 1.33 μmol/L L-arabinose (Merck, Darmstadt, Germany).

### 2.3 Growth curves

Growth curves were performed in 1 L Erlenmeyer flasks containing either 250 or 500 mL of LB medium and incubated at 37 °C under constant agitation at 120 or 180 rpm. Cultures were inoculated at a 1:1000 dilution using stationary-phase overnight cultures grown at 37 °C. Bacterial growth was monitored by measuring optical density at 600 nm (OD_600_) and colony-forming units per milliliter (CFU/mL) every hour during the first 10 h of incubation and again at 24 h. For CFU determination, cultures were serially diluted in 10-fold steps, and 5 μL of each dilution was spotted onto LB agar plates followed by incubation for 14 h at 37 °C before colony counting. All experiments were performed using three independent biological replicates (*n* = 3).

### 2.4 OMV enrichment and recovery: standard protocol

OMVs were isolated following established protocols with some modifications (Liu et al., 2016b; Nevermann et al., 2019; Fuentes et al., 2025; Parra et al., 2025). *S.* Typhi strains were cultured in LB medium at 37 °C under the standardized baseline conditions (1 L total culture volume, obtained by pooling four 250 mL cultures grown in parallel in 1 L Erlenmeyer flasks, 120 rpm, 14 h, OD_600_ ≈ 1.0) to ensure comparable starting material across purification methods. Bacterial cells were removed by centrifugation at 5400 × *g* for 10 min at 4 °C. The supernatant was collected and filtered through 0.45 μm pore-size membranes (Nalgene™, Gibco, Thermo Fisher Scientific, Waltham, MA, United States) to remove residual cells and large debris. The clarified supernatant (1 L) was then concentrated to a retentate volume of approximately 15–20 mL (∼50–67-fold volumetric concentration factor, over a processing time of approximately 40 min) using either Ultracel™ 100 kDa ultrafiltration discs (Normal Flow Filtration, NFF; Amicon™ Bioseparations) or a Vivaflow^®^ 200 tangential flow filtration (TFF) system equipped with 100 kDa Hydrosart^®^ membranes (Sartorius, 200 cm^2^ surface area). For TFF processing, the system was operated according to the manufacturer’s instructions, maintaining operating pressures below 3 bar and filtration flow rates between 20 and 25 mL/min.

Concentrated samples were subsequently subjected to ultracentrifugation using a Sorvall™ WX ultracentrifuge (Thermo Fisher Scientific) with an AH-629 rotor at 150,000 × *g* for 3 h at 4 °C. Following centrifugation, the supernatant was discarded, and the OMV-containing pellet was resuspended in 1 mL of PBS (Gibco, Thermo Fisher Scientific, Waltham, MA, United States). The OMV suspensions were subsequently filtered through 0.22 μm pore-size filters and stored at −80 °C until further use.

### 2.5 OMV enrichment and recovery: ultracentrifugation-free protocol

An alternative, ultracentrifugation-free workflow was established by combining TFF concentration, discontinuous diafiltration, lyophilization, and reconstitution. *S.* Typhi cultures were prepared under the standardized conditions described above to obtain 1 L of total culture volume (four 250 mL cultures grown in parallel).

Following cell removal by centrifugation (5,400 × g, 10 min, 4 °C) and primary clarification through 0.45-μm-pore-size membranes, the clarified supernatant (1 L) was pre-concentrated to approximately 100 mL (10-fold volumetric concentration) using the Vivaflow^®^ 200 TFF system (100 kDa Hydrosart^®^ membrane) operated under the same pressure and flow rate parameters described in the standard protocol. Without removing the retentate from the filtration circuit, discontinuous diafiltration was performed by adding a total of 4 L of exchange solution (either 0.5% w/v NaCl or 0.5% w/v sucrose in sterile distilled water) in sequential volumes. The diafiltered retentate was subsequently reduced to a final volume of 15–20 mL (∼50–67-fold overall concentration factor).

Following diafiltration and final volume reduction, the OMV-enriched preparations were transferred to 50-mL conical tubes. The tube openings were covered with parafilm perforated with small holes to allow water-vapor release during lyophilization. Samples were initially frozen in the cold trap of an INOFD-10PU lyophilizer (Innova Bio-Meditech, Qingdao, China) at −70 °C for 2 h. The frozen samples were then transferred to the lyophilizer shelves, and a vacuum of 5–15 Pa was applied. Lyophilization was performed as a single drying cycle for 24–48 h. Separate primary- and secondary-drying stages were not independently programmed or monitored. The cycle was terminated when no visible ice or residual liquid remained. The lyophilized preparations were stored at 4 °C until reconstitution.

Lyophilized OMV preparations were reconstituted in 1 mL of sterile PBS solution by gentle inversion. The same final resuspension solution and volume were used for all subsequent downstream workflow comparisons, independently of the processing workflow, thereby standardizing the final sample matrix and volume for downstream comparisons. The resulting OMV suspensions were passed through 0.22-μm-pore-size filters and stored at −80 °C until further use.

For downstream applications potentially affected by residual NaCl or sucrose, including transmission electron microscopy and SDS–PAGE, reconstituted OMVs were additionally washed with sterile distilled water using 100-kDa molecular-weight-cutoff centrifugal filtration devices (Amicon™, Merck, Darmstadt, Germany) to remove excess NaCl or sucrose. The retained OMVs were subsequently resuspended in PBS (Gibco, Thermo Fisher Scientific, Waltham, MA, United States).

### 2.6 Experimental design for OMV workflow comparison

To compare conventional and ultracentrifugation-free OMV processing strategies, OMVs were prepared from *S.* Typhi cultures grown under the selected low-agitation condition described above. Four processing workflows were evaluated. The conventional control condition consisted of OMVs concentrated from clarified culture supernatants and subsequently recovered by ultracentrifugation without lyophilization (TFF/UC+Lyo−). A second conventional condition consisted of OMVs obtained by the same ultracentrifugation-based protocol and then subjected to lyophilization (TFF/UC+Lyo+), allowing the effect of lyophilization to be evaluated independently of the TFF/diafiltration workflow. Two ultracentrifugation-free workflows were then evaluated. In these conditions, clarified and filtered culture supernatants were concentrated by tangential flow filtration (TFF), subjected to diafiltration using either 0.5% NaCl or 0.5% sucrose as the exchange solution, and subsequently lyophilized. These conditions are referred to as TFF/DF-NaCl+Lyo+ and TFF/DF-sucrose+Lyo+, respectively. Diafiltration was performed using four volumes of exchange solution relative to the original culture volume. To ensure comparability between workflows, all OMV preparations were finally resuspended in the same volume (1 mL) of PBS, independently of whether they were obtained by ultracentrifugation or diafiltration and whether they were analyzed before or after lyophilization. The resulting OMV preparations were compared using complementary structural, biochemical, physicochemical, and functional readouts, including transmission electron microscopy, SDS-PAGE, nanoparticle tracking analysis, protein and lipid quantification, proteinase K protection assays, and mCherry fluorescence measurements. This design allowed the effect of lyophilization to be evaluated within the ultracentrifugation-based branch, while the TFF/diafiltration/lyophilization conditions were evaluated as integrated ultracentrifugation-free workflows.

The inclusion of both TFF/UC+Lyo− and TFF/UC+Lyo+ allowed the effect of lyophilization to be evaluated within the ultracentrifugation-based branch. In contrast, because both diafiltration conditions were subsequently lyophilized, the TFF/DF-NaCl+Lyo+ and TFF/DF-sucrose+Lyo+ conditions were evaluated as complete integrated workflows rather than as a factorial assessment of the independent effects of diafiltration and lyophilization.

### 2.7 Assessment of residual viable bacteria in OMV preparations

To confirm the absence of residual viable bacteria, aliquots of clarified and filtered culture supernatants, as well as final OMV preparations, were plated on LB agar and incubated at 37 °C for 24–48 h. When appropriate, plates were also supplemented with the corresponding antibiotic used for the producing strain. OMV preparations were considered free of viable bacterial contamination when no colony growth was detected after incubation. Only bacteria-free OMV preparations were used for downstream biochemical, physicochemical, and functional assays.

### 2.8 Nanoparticle tracking analysis of OMV preparations

Particle concentration and size distribution of OMV preparations were determined by nanoparticle tracking analysis (NTA). Because NTA detects light-scattering particles independently of biological origin, particle-associated signals were interpreted cautiously and not assumed to represent exclusively vesicular material. Each OMV fraction was measured using a NanoSight^®^ NS300 instrument and analyzed with NTA software version 3.4, build 3.4.4 (Malvern Panalytical, Malvern, United Kingdom). Each analysis was carried out using a 488 nm laser and the following parameters: camera level 12, detection threshold 5, room temperature, water viscosity, three 30-s video acquisitions. Prior to measurement, samples were diluted in sterile PBS (Gibco, Thermo Fisher Scientific, Waltham, MA, United States) to fall within the optimal particle concentration range recommended by the manufacturer. The data were summarized in tables and represented graphically as particles/mL versus particle size.

When required, particle numbers were normalized to the bacterial biomass of the corresponding culture and expressed as particles/CFU. In addition, protein- and lipid-associated signals were normalized to particle number to estimate the relative protein or lipid content per particle. All NTA measurements were performed using OMV preparations obtained from independent biological replicates, and data are reported as mean ± SEM unless otherwise indicated.

### 2.9 OMV quantification

Protein concentration was measured using a Pierce™ BCA Protein Assay Kit (Thermo Fisher Scientific, Waltham, MA, United States), while lipid quantification was performed using the fluorescent probe FM4-64 (Thermo Fisher Scientific, Waltham, MA, United States), as previously described (Nevermann et al., 2019; Marchant et al., 2021; Marchant et al., 2024; Fuentes et al., 2025). Particle concentration was determined by nanoparticle tracking analysis (NTA), as described above. Relative OMV production was assessed by normalizing protein concentration, FM4-64 fluorescence signal, and particle concentration to the CFU/mL of the corresponding bacterial culture. Additionally, protein-to-particle and lipid-to-particle ratios were calculated by dividing protein concentration or FM4-64 fluorescence values by the corresponding particle concentration obtained by NTA. For downstream assays requiring matched OMV input, preparations were normalized according to protein concentration, as specified for each assay. In contrast, mCherry fluorescence measurements were performed using equal preparation volumes.

### 2.10 Transmission electron microscopy (TEM) and OMV size measurement

OMV samples were adsorbed onto Formvar-coated slot grids (EMS, Hatfield, PA, United States) and negatively stained with 1% aqueous uranyl acetate (EMS, Hatfield, PA, United States) for 1 min. Following previously described protocols (Nevermann et al., 2019), the grids were examined using a Talos F200C G2 transmission electron microscope (Thermo Fisher Scientific, MA, United States). OMV size was determined from TEM images obtained from three fields per biological replicate, as described previously (Deatherage et al., 2009; Marchant et al., 2024; Fuentes et al., 2025). For each biological replicate, at least 100 OMVs were measured across the three images using ImageJ software (version 1.54p, National Institutes of Health, Bethesda, MD, United States), and vesicle size is reported as diameter. Only vesicles with visible membrane boundaries were included in the analysis, while electron-dense debris and non-vesicular structures were excluded. In samples containing OMV aggregates, individual vesicles with visible boundaries were measured independently.

### 2.11 Zeta potential determination

Zeta potential determination was performed following the protocol previously described by Marchant et al. (2021) and Marchant et al. (2024). Briefly, an aliquot of previously extracted OMVs corresponding to 50 μg of protein was diluted to a final volume of 1 mL in sterile ultrapure water (UPW) (Winkler, Lampa, Chile). Zeta potential measurements were conducted at room temperature (25 °C) using an MPT-Z multipurpose titrator coupled to a Zetasizer Nano series instrument (Malvern Instruments, Malvern, United Kingdom). The device was equipped with a 633 nm Helium–Neon laser as the light source, and measurements were performed at a detection angle of 173.13° in aqueous medium.

### 2.12 SDS-PAGE

SDS-PAGE was performed as described previously (Nevermann et al., 2019; Fuentes et al., 2025). OMVs, corresponding to 30 μg of protein, were resolved on SDS-PAGE gels and stained with One-Step Blue Protein Gel Stain (Biotium, Fremont, CA, United States) for visualization.

### 2.13 Western blot assays

OMV samples (30 μg of protein) were resolved by SDS-PAGE and transferred onto nitrocellulose membranes. To confirm equal protein loading, membranes were stained with Ponceau S (Thermo Fisher Scientific, Waltham, MA, United States) as previously described (Sander et al., 2019; Fuentes et al., 2025), and subsequently rinsed with distilled water. For the detection of FLAG or flagellin, membranes were incubated with either a primary mouse anti-FLAG M2 monoclonal antibody (1:5,000, Sigma-Aldrich, St. Louis, MO, United States) or a primary rabbit anti-flagellin polyclonal antibody (1:10,000, Abcam, Cambridge, United Kingdom), followed by the appropriate horseradish peroxidase (HRP)-conjugated secondary antibody: goat anti-mouse IgG (1:5,000, Sigma-Aldrich, St. Louis, MO, United States) or goat anti-rabbit IgG (1:5,000, Sigma-Aldrich, St. Louis, MO, United States), respectively. As a loading control, identical samples were run on SDS-PAGE and stained with One-Step Blue Protein Gel Stain (Biotium, Fremont, CA, United States). Protein detection in all cases was performed using enhanced chemiluminescence (Clarity Western ECL Substrate, Bio-Rad, Hercules, CA, United States).

### 2.14 Epithelial cell culture

The human colorectal adenocarcinoma cell line HT-29 (ECACC 91072201, European Collection of Authenticated Cell Cultures) was cultured in Dulbecco′s Modified Eagle Medium (DMEM, High Glucose, Gibco, Thermo Fisher Scientific, Waltham, MA, United States) containing 4.5 g/L D-glucose, L-glutamine, and 110 mg/L sodium pyruvate. The medium was supplemented with 10% (v/v) fetal bovine serum (FBS) (Gibco, Thermo Fisher Scientific, Waltham, MA, United States) and 1 × antibiotic–antimycotic (Gibco, Thermo Fisher Scientific, Waltham, MA, USA). The cells were maintained at 37 °C in a humidified atmosphere with 5% CO_2_. The culture medium was refreshed every 3–4 days, and the cells were subcultured weekly using 0.25% trypsin–EDTA (0.53 mM) (Gibco, Thermo Fisher Scientific, Waltham, MA, United States).

### 2.15 Fluorescent labeling of OMVs with DiO

OMVs (100 μg total protein) were fluorescently labeled with 3,3′-dioctadecyloxacarbocyanine perchlorate (DiO; Thermo Fisher Scientific, Waltham, MA, United States) using a 1 mM stock solution to achieve a final concentration of 10 μM. Labeling was performed by incubation at 37 °C for 20 min, as previously described (Turner et al., 2018; Fuentes et al., 2025). Excess unincorporated dye was removed by washing with sterile phosphate-buffered saline (PBS; Gibco, Thermo Fisher Scientific, Waltham, MA, United States) using a 100 kDa molecular weight cutoff (MWCO) filtration column (Amicon™, Merck, Darmstadt, Germany). For cell-exposure assays, HT-29 cells were maintained in 0.22 μm-filtered phenol red-free DMEM supplemented with 10% fetal bovine serum. For fluorescence-quenching experiments, trypan blue was added at a 1:1 volume ratio immediately prior to acquisition and incubated for 5 min before flow cytometry analysis.

### 2.16 Flow cytometry analysis of OMV-associated and quenching-resistant fluorescence in HT-29 cells

HT-29 cells (ECACC 91072201) were incubated at a density of 2 × 10^6^ cells in 1 mL of filtered DMEM containing 100 μg/mL of DiO-labeled OMVs for 0, 30 min, 1, 2, 3, or 5 h at 37 °C in a humidified 5% CO_2_ atmosphere. Untreated cells were included as negative controls. Following incubation, cells were washed with PBS to reduce non-associated OMVs prior to flow cytometry analysis. Samples identified as “AT” were treated with trypan blue immediately before acquisition to quench extracellular fluorescence and obtain a trypan blue-resistant fluorescence readout. Cells were analyzed using a FACSAria III flow cytometer (Becton Dickinson, San Jose, CA, United States). Cellular debris was excluded based on forward- and side-scatter (FSC/SSC) parameters, and singlet cells were selected using FSC-A versus FSC-H gating. DiO-derived fluorescence was detected in the FITC channel (FITC-A). Additional controls consisting of DiO-only washing fractions processed in parallel with OMV preparations were included to evaluate potential background fluorescence derived from free dye. Cells exhibiting fluorescence intensity above the threshold defined by control samples were considered DiO-positive, and the percentage of DiO-positive cells was used as the reported parameter. Approximately 20,000 events were acquired per sample whenever possible. Data were analyzed using FlowJo software version 10.

### 2.17 Confocal microscopy analysis of HT-29 cells exposed to *S*. Typhi OMVs labeled with DiO

HT-29 cells (ECACC 91072201) were cultured on glass coverslips placed in 24-well plates. Cells were seeded at a density of 2 × 10^5^ cells per well and incubated overnight at 37 °C in a 5% CO_2_ atmosphere to allow adherence and initial growth. Following incubation, cells were washed three times with phosphate-buffered saline (PBS; Gibco, Thermo Fisher Scientific, Waltham, MA, United States) to remove residual medium. Cells were then treated with 50 μg/mL of DiO-labeled OMVs (quantified by protein content). Treatments were performed for 3 h at 37 °C, followed by thorough PBS washes to eliminate unbound OMVs or excess dye. To preserve cellular structures, cells were fixed with 4% paraformaldehyde (PFA; Merck, Darmstadt, Germany) for 20 min at room temperature and subsequently washed three times with PBS. Nuclei were stained with Hoechst 33342 (Thermo Fisher Scientific, Waltham, MA, United States) at 3 μg/mL for 15 min. When required, the plasma membrane was stained with Alexa Fluor^®^ 555-conjugated wheat germ agglutinin (WGA; Thermo Fisher Scientific, Waltham, MA, United States) at 5 μg/mL for 15 min in the dark. Coverslips were mounted onto glass slides using Fluoromount-G (Thermo Fisher Scientific) to preserve fluorescence. Confocal imaging was performed using a Leica TCS SP8 confocal microscope (Leica Microsystems, Wetzlar, Germany) equipped with 405 nm (Hoechst), 488 nm (DiO), and 552 nm (Alexa Fluor^®^ 555) lasers. The photomultiplier tube (PMT) detection ranges were set to 410–483 nm for the Hoechst channel, 505–547 nm for the DiO channel, and 587–726 nm for the WGA channel. For comparative imaging, laser power, gain, offset, pinhole, scanning speed, and detector settings were kept constant across conditions within each experiment. Image acquisition and processing were carried out using LAS X software (version 3.1.5.16308). Image deconvolution was performed using Huygens Essential software (version 26.04.0p1, Scientific Volume Imaging, Hilversum, The Netherlands) using a theoretical point spread function (PSF), the Standard deconvolution mode, and the Classic Maximum Likelihood Estimation (MLE) algorithm with optimized iteration mode and a maximum of 30 iterations.

### 2.18 Bioassay for determining β-lactamase activity

The bioassay was performed following the protocol previously described by Nevermann et al. (2019). Briefly, *S.* Typhi strain expressing β-lactamase (*bla* gene) from a single chromosomal copy was constructed by chromosomal insertion of the plasmid pNFB9, which carries the *bla* gene, the Gifsy-1 phage attachment site (*att*_*BG*1_), and the corresponding integrase gene *int* (Lemire et al., 2008). This plasmid integrates into the naturally vacant *att*_*BG*1_ site of the *S.* Typhi chromosome, resulting in the strain *S.* Typhi *att*_*BG*1_::pNFB9 (Lac^–^ Amp*^R^*). OMVs obtained from this strain were serially diluted two-fold and 5 μL of OMV preparations from equal starting volumes were spotted onto a lawn of the reporter strain *S.* Typhimurium ATCC14028s Δ*ompD*::MudJ (Lac^+^ Amp*^S^*), prepared from an overnight culture and previously spread on LB agar plates supplemented with ampicillin (50 μg/mL, AppliChem GmbH, Darmstadt, Germany) and X-gal (5-bromo-4-chloro-3-indolyl-β-D-galactopyranoside, 40 μg/mL, Merck, Darmstadt, Germany). The presence of functional β-lactamase was evidenced by the appearance of blue satellite colonies surrounding the OMV spots. As a control, the same OMV preparations were spotted onto LB agar plates lacking antibiotics and indicator.

### 2.19 Fluorescence activity assay for mCherry in OMVs

The fluorescence activity of mCherry in OMV preparations was measured using a Synergy H1M microplate reader (BioTek, Winooski, VT, United States), as previously described (Fuentes et al., 2025). Briefly, 200 μL of each OMV preparation were loaded into black clear-bottom 96-well plates. Samples originated from equivalent starting culture volumes and were analyzed using equal preparation volumes; lyophilized samples were reconstituted to the same final volume before measurement. Fluorescence values were therefore compared on a preparation-volume basis and were not normalized to particle number or total protein. Accordingly, this assay reports the total detectable mCherry-associated fluorescence in the recovered preparation rather than fluorescence per particle.

For endpoint fluorescence readings, the excitation wavelength was set to 587 nm, the optimal value for mCherry excitation, while the emission wavelength was recorded at 630 nm. Although the optimal emission wavelength for mCherry is 610 nm, it was adjusted to 630 nm due to the spectral overlap limitations of the plate reader. As a negative control, OMVs derived from the parental WT strain lacking mCherry expression were previously shown to exhibit no detectable fluorescence under these conditions (Fuentes et al., 2025).

### 2.20 Proteinase K protection assay

To assess membrane-dependent protection of OMV-associated mCherry-FLAG cargo, 30 μg of OMVs (quantified by protein content) were treated with 0.5 μg/mL of proteinase K (Thermo Fisher Scientific, Waltham, MA, United States) at 37 °C for 30 min, either in the presence or absence of 1% SDS (Winkler, Lampa, Chile). Because mCherry was fused to a FLAG tag, its presence in proteinase K-treated OMVs was evaluated by Western blot using an anti-FLAG antibody, as previously described (Muralinath et al., 2011; Fantappie et al., 2014; Fuentes et al., 2025).

### 2.21 OMV storage

To minimize the effects of repeated freeze–thaw cycles on OMV stability (Alves et al., 2016), OMV preparations were aliquoted into equal volumes in microcentrifuge tubes according to storage condition and downstream experimental use. Samples were stored at 4, −20, or −80 °C, whereas additional aliquots were subjected to lyophilization as described above. Following lyophilization, tubes were visually inspected to confirm the absence of residual ice, sealed immediately after drying, and stored at 4 °C protected from environmental moisture until reconstitution. Lyophilized OMVs were reconstituted in 1 mL of sterile PBS immediately before use. OMV preparations stored at 4, −20, −80 °C, or under lyophilized conditions were evaluated at predefined time points for up to 120 days.

### 2.22 Statistical analysis

Statistical analyses were performed using GraphPad Prism 8. Data are presented as mean ± standard error of the mean (SEM) unless otherwise indicated. The number of independent biological replicates is indicated in each figure legend. For comparisons between two experimental groups, statistical significance was assessed using an unpaired two-tailed Student’s *t*-test. For comparisons involving more than two groups, one-way analysis of variance (ANOVA) followed by Tukey’s multiple-comparison *post-hoc* test was used. For analyses involving particle size distributions or zeta potential measurements, individual measurements were used to visualize distribution patterns, whereas statistical comparisons were performed using replicate-level summary values whenever applicable, in order to avoid overestimating statistical independence. When distributional data did not meet the assumptions required for parametric testing, non-parametric analysis using the Kruskal–Wallis test followed by Dunn’s multiple-comparison test was applied. For time-course cell-associated and trypan blue-resistant fluorescence experiments, comparisons between experimental conditions were performed at matched time points using the appropriate two-group test or multiple-comparison analysis, as indicated in the corresponding figure legends. Differences were considered statistically significant when *p* < 0.05. Statistical significance is indicated in the figures using asterisks or distinct letters, as specified in each figure legend.

## 3 Results

### 3.1 Culture agitation differentially affects protein-, lipid-, and particle-associated recovery

**FIGURE 2:**
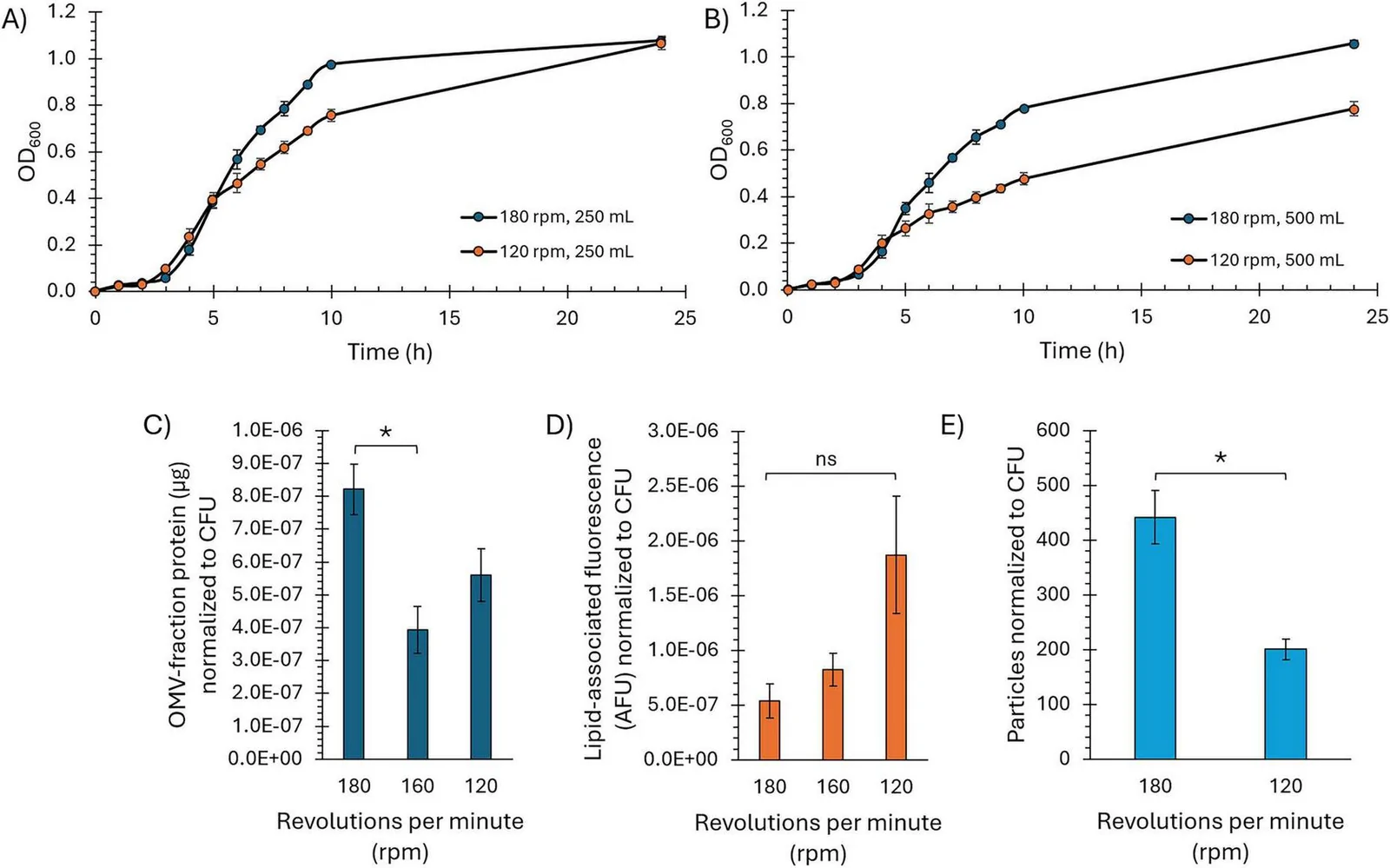
Effect of culture volume and agitation speed on *S*. Typhi growth and apparent OMV-associated recovery. Growth kinetics of *S.* Typhi cultured in 1 L Erlenmeyer flasks containing either 250 mL **(A)** or 500 mL **(B)** of LB medium and incubated at 37 °C under agitation at 120 or 180 rpm. Bacterial growth was monitored by measuring OD_600_ hourly during the first 10 h and again at 24 h. **(C)** Apparent OMV-associated recovery quantified as total protein content and normalized to viable bacterial density (CFU/mL) in cultures grown at 120, 160, or 180 rpm. **(D)** Apparent OMV-associated lipid recovery determined using FM4-64 fluorescence and normalized to CFU/mL under the same agitation conditions. Fluorescence values are expressed as arbitrary fluorescence units (AFU). **(E)** Particle-associated recovery determined by nanoparticle tracking analysis and normalized to CFU/mL for OMVs obtained from cultures grown at 120 or 180 rpm. Data are presented as mean ± SEM from three independent biological replicates. Statistical analysis was performed using one-way ANOVA followed by Tukey’s multiple-comparison test for panels **(C,D)**, and an unpaired two-tailed Student’s *t*-test for panel **(E)**. Statistically significant differences are indicated (*p < 0.05).

To determine whether upstream culture conditions affected bacterial growth and OMV-associated recovery, *S.* Typhi was cultured using different flask filling volumes and agitation speeds. Cultures reached similar final densities, although the 500 mL cultures entered exponential growth later than the 250 mL cultures (Figures 2A,B). Therefore, the 250 mL culture volume was selected to evaluate agitation-dependent effects on the recovered OMV-enriched fraction.

OMV-associated recovery was then evaluated using complementary quantitative readouts. When normalized to viable bacterial density, protein-associated recovery differed across agitation conditions, with higher values detected at increased agitation speed (Figure 2C). In contrast, lipid-associated fluorescence measured with FM4-64 and normalized to CFU did not differ significantly among agitation conditions (Figure 2D). NTA-detected particle recovery was also higher at 180 rpm than at 120 rpm (Figure 2E). However, protein-to-particle and lipid-to-particle ratios did not differ significantly between these conditions (Supplementary Figures 2A,B).

Thus, increased agitation raised protein- and particle-associated recovery readouts without a corresponding increase in lipid-associated fluorescence. These results prompted evaluation of whether the higher apparent recovery was accompanied by changes in preparation morphology and contaminant carryover.

### 3.2 Low-agitation culture reduces flagellin carryover and is associated with higher epithelial cell-associated fluorescence

**FIGURE 3:**
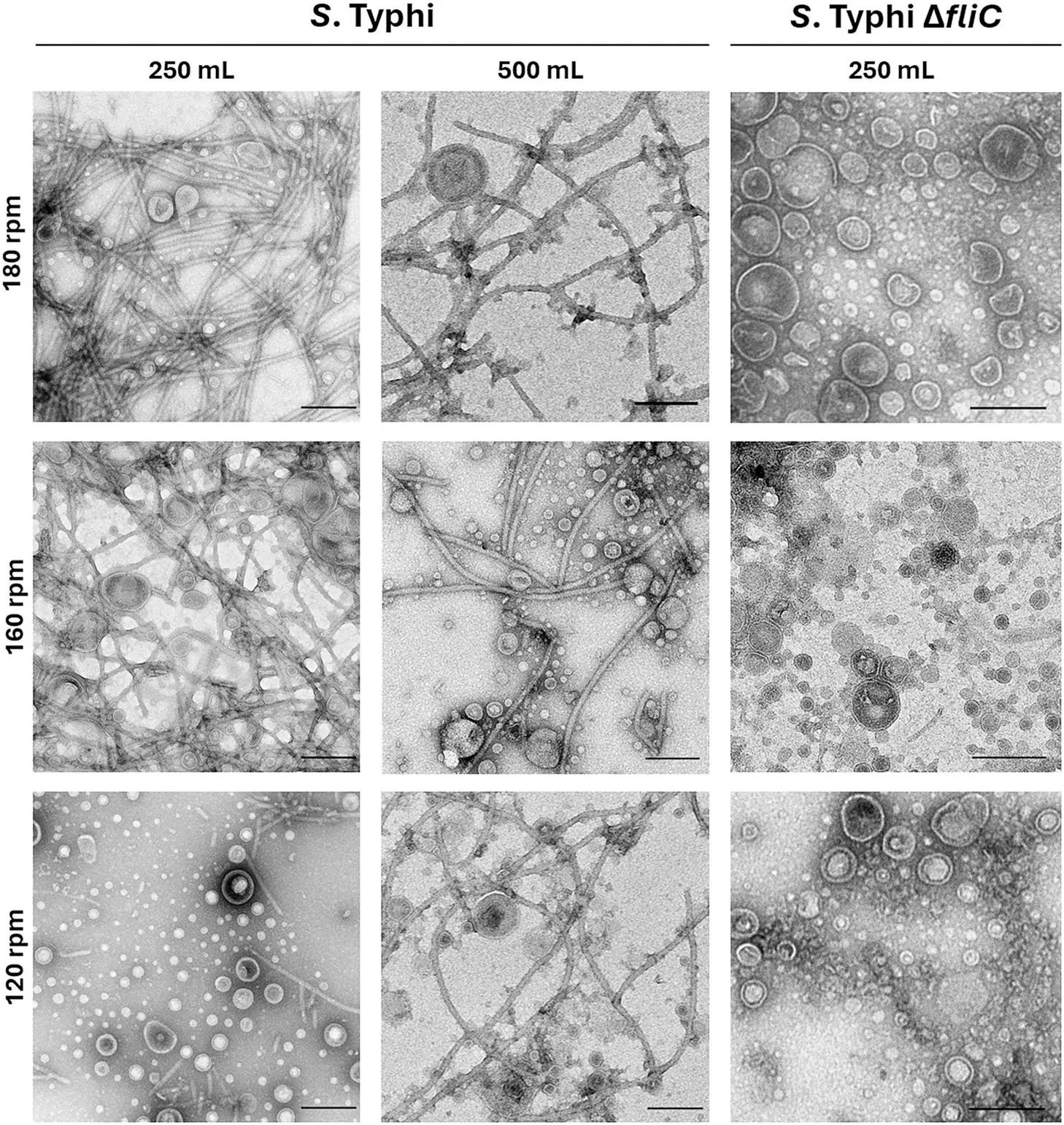
Effect of agitation speed and culture volume on *S*. Typhi OMV morphology and filamentous material co-isolation. OMV preparations were obtained from *S.* Typhi WT cultures grown in 1 L Erlenmeyer flasks containing either 250 mL or 500 mL of LB medium and incubated at 37 °C under agitation at 120, 160, or 180 rpm. OMVs from an isogenic Δ*fliC* mutant strain grown under the same agitation conditions in 250 mL cultures were included as a genetic control for flagellin-associated filamentous structures. Preparations were analyzed by transmission electron microscopy (TEM). Scale bars represent 200 nm. Images from 250 mL cultures were acquired at 45,000 × magnification, whereas images from 500 mL cultures were acquired at 28,000 × magnification. Representative images from three independent biological replicates are shown for each condition.

Because higher recovery could reflect co-isolation of non-vesicular material, we examined the morphology and flagellin-associated carryover of preparations generated under different agitation conditions. TEM revealed abundant filamentous material in WT preparations obtained at 180 rpm, whereas this material was markedly reduced at 120 rpm, particularly in 250 mL cultures (Figure 3). Preparations obtained at 160 rpm showed an intermediate and volume-dependent phenotype. Filamentous structures were not evident in preparations from the Δ*fliC* strain, supporting their assignment as flagellar or flagellin-associated material.

NTA analysis showed that all preparations contained particles within the expected nanometric size range for bacterial OMVs. In contrast, particle concentration showed marked numerical differences among conditions, with higher particle numbers generally observed at 180 rpm and lower recovery in the Δ*fliC* background, particularly at 120 rpm (Table 3). Because NTA detects light-scattering particles without establishing their vesicular identity, the higher particle counts at 180 rpm may include contributions from co-isolated filamentous or non-vesicular material. These observations suggest that high agitation increases the recovery of particle-associated material but also promotes co-isolation of flagellin-containing structures, whereas low-agitation culture improves the morphological definition of *S.* Typhi OMV preparations.

**TABLE 3:** Nanoparticle tracking analysis of OMV preparations obtained from *S*. Typhi WT and Δ*fliC* strains grown under different agitation conditions.

OMVs source	Revolutions per min (rpm)	Volume (mL)	Mean size (nm)	Mode (nm)	Concentration (particles/mL)
*S*. Typhi	180 rpm	250 mL	135.5 ± 2.2	112.7 ± 3.0	1.07 × 10^12^ ± 6.98 × 10^10^
*S.* Typhi	120 rpm	250 mL	140.6 ± 4.6	105.3 ± 4.2	3.92 × 10^11^ ± 1.93 × 10^10^
*S.* Typhi Δ*fliC*	180 rpm	250 mL	123.4 ± 1.4	99.8 ± 8.5	4.93 × 10^11^ ± 1.20 × 10^10^
*S.* Typhi Δ*fliC*	120 rpm	250 mL	130.2 ± 2.5	105.4 ± 7.2	1.21 × 10^11^ ± 1.10 × 10^10^

OMVs were obtained from *S.* Typhi WT and isogenic Δ*fliC* cultures grown in 1 L Erlenmeyer flasks containing 250 mL of LB medium and incubated at 37 °C under agitation at either 120 or 180 rpm. Particle size distribution and concentration were determined by nanoparticle tracking analysis (NTA). Mean particle diameter, modal diameter, and particle concentration are reported for each condition. Across all preparations, mean and modal diameters remained within the expected nanometric size range for bacterial OMVs, whereas particle concentration showed marked numerical variation according to agitation speed and strain background. Data are presented as mean ± SEM from three independent biological replicates.

**FIGURE 4:**
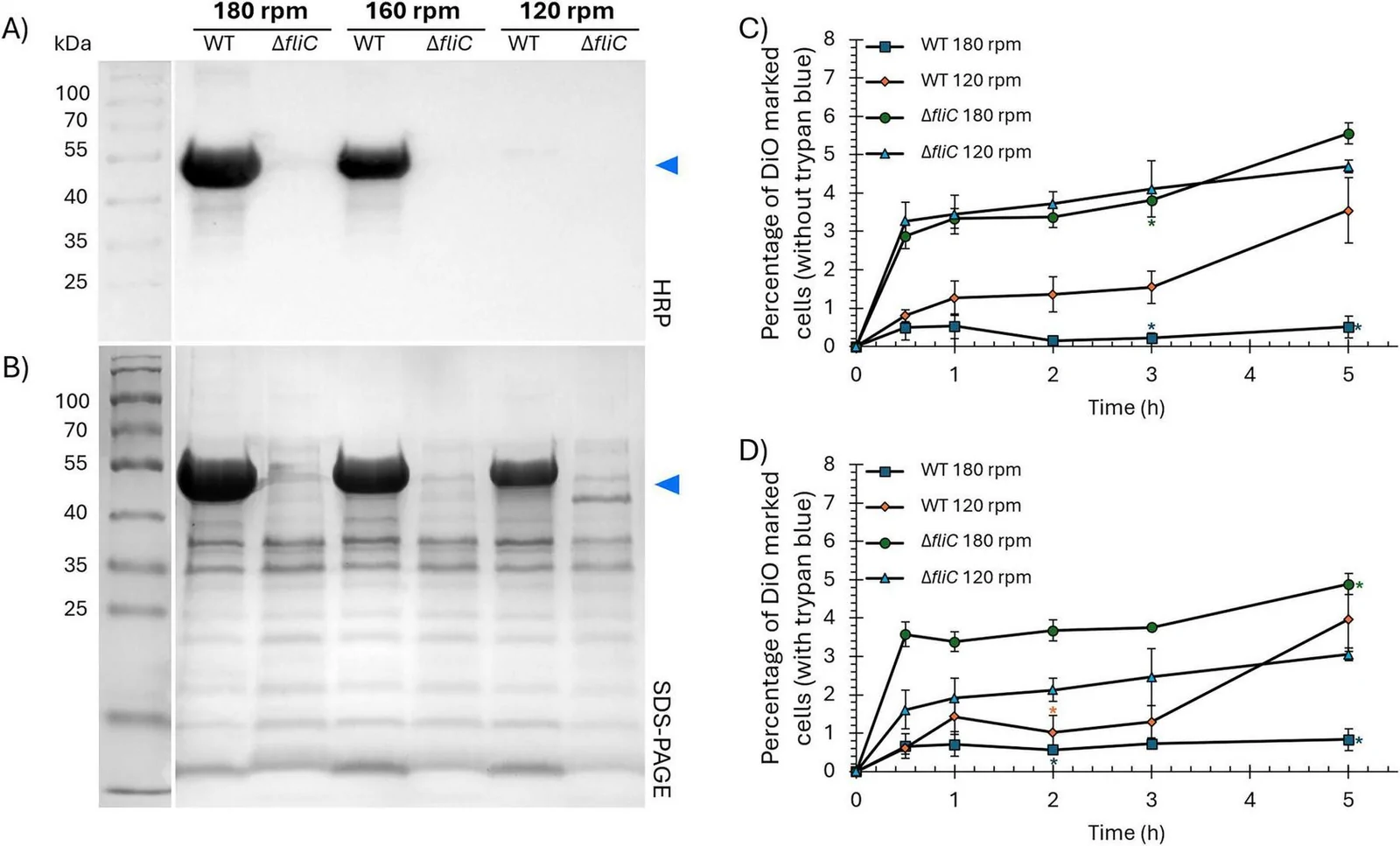
Low-agitation culture reduces flagellin carryover and is associated with increased epithelial cell interaction of *S.* Typhi OMVs. **(A)** Western blot analysis of OMV preparations obtained from *S.* Typhi WT and isogenic Δ*fliC* strains grown in 250 mL of LB medium at 37 °C under agitation at 180, 160, or 120 rpm. Flagellin was detected using a polyclonal anti-flagellin antibody. Each lane was loaded with 30 μg of OMVs, quantified by total protein content. The blue arrowhead indicates the expected ∼49 kDa flagellin band, which was detected in WT-derived preparations with stronger signal at higher agitation speeds and minimal or reduced signal at 120 rpm, but was not detected in Δ*fliC*-derived preparations. **(B)** SDS-PAGE analysis of the same OMV samples stained with One-Step Blue Protein Gel Stain to evaluate overall protein loading and profile. The blue arrowhead indicates the position of the flagellin band. **(C,D)** Flow cytometry analysis of HT-29 cells exposed to 100 μg/mL DiO-labeled OMVs derived from WT or Δ*fliC* strains grown at 120 or 180 rpm (gating strategy shown in Supplementary Figure 5). Total cell-associated fluorescence was measured in the absence of trypan blue quenching **(C)**, whereas trypan blue-resistant fluorescence was used as an uptake-associated readout enriched for internalized OMVs **(D)**. Data are presented as mean ± SEM from three independent biological experiments. Different lowercase letters indicate statistically significant differences; groups sharing at least one letter are not significantly different, as determined by two-way ANOVA followed by Tukey’s multiple-comparison test.

To biochemically validate the presence of flagellin in these preparations, OMVs from WT and Δ*fliC* strains grown at 120, 160, and 180 rpm were analyzed by Western blot using an anti-flagellin antibody. WT preparations showed an agitation-dependent flagellin signal, with the strongest signal at 180 rpm, reduced signal at 160 rpm, and minimal signal at 120 rpm, whereas no signal was detected in Δ*fliC*-derived preparations (Figure 4A). SDS-PAGE showed broadly similar total-protein loading patterns (Figure 4B).

To determine whether the agitation-dependent differences in preparation morphology and flagellin carryover were accompanied by differences in epithelial cell-associated fluorescence, HT-29 intestinal epithelial cells were incubated with DiO-labeled OMV preparations. Cell-associated fluorescence was measured over time by flow cytometry in the absence or presence of trypan blue quenching. WT preparations obtained at 120 rpm generated higher cell-associated and trypan blue-resistant fluorescence than those obtained at 180 rpm, whereas Δ*fliC*-derived preparations were less affected by agitation (Figures 4C,D). These results establish an association between high agitation, increased flagellin carryover, and reduced epithelial cell-associated fluorescence. Based on its lower flagellin carryover, improved morphological definition, and retained cell-associated fluorescence, 120 rpm with a 250 mL filling volume was selected for subsequent processing experiments.

### 3.3 Tangential flow filtration (TFF) preserves the measured preparation attributes relative to normal-flow filtration (NFF) preconcentration

Using the selected upstream condition, we compared TFF and NFF as preconcentration steps before ultracentrifugation and assessed morphology, protein profile, apparent recovery, and NTA-detected particle characteristics.

**FIGURE 5:**
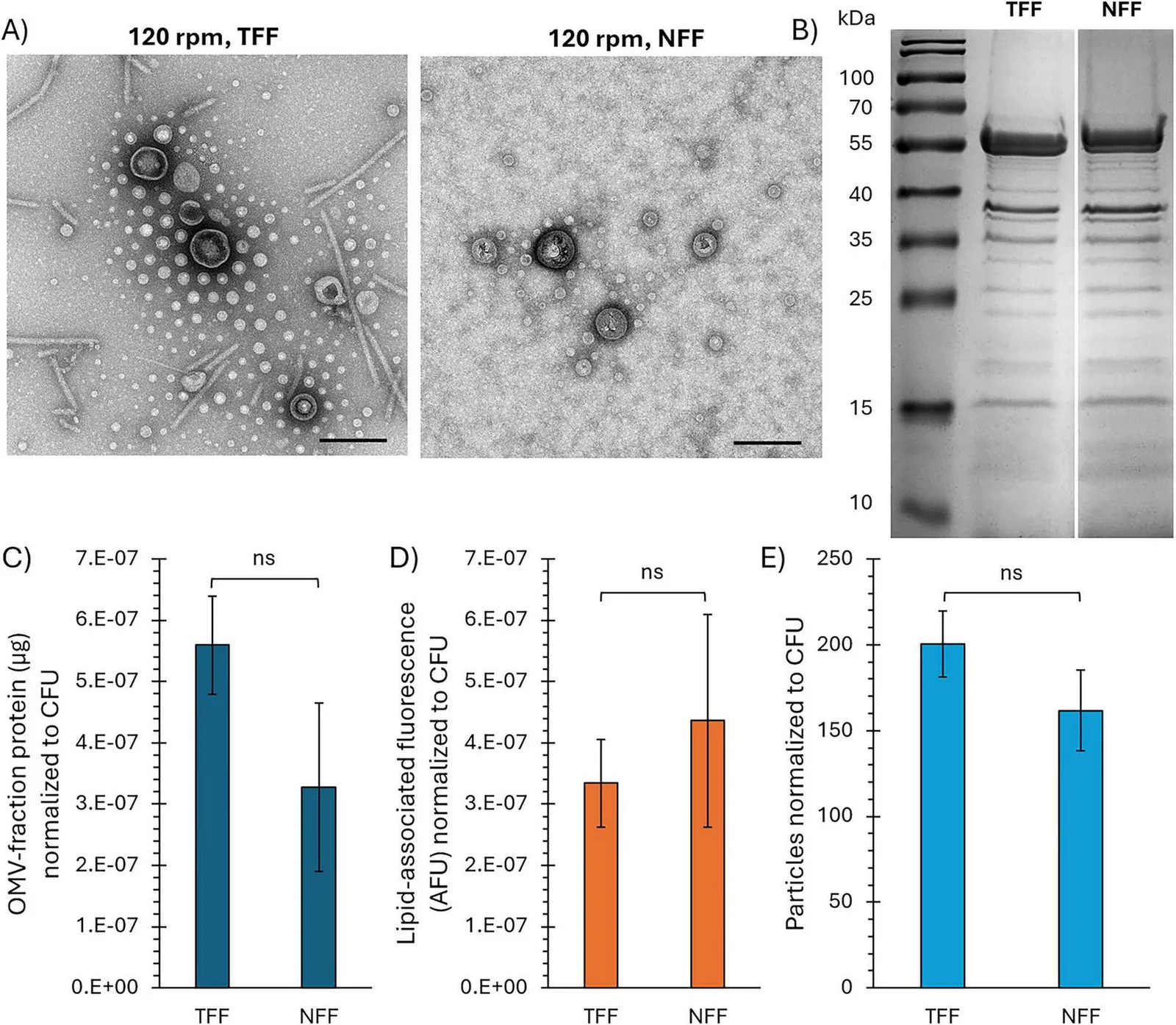
Comparative evaluation of tangential flow filtration and normal-flow filtration as preconcentration strategies for *S.* Typhi OMV processing. OMVs were obtained from *S.* Typhi cultures grown under the selected low-agitation condition, 120 rpm in 250 mL culture volumes. Clarified and filtered culture supernatants were concentrated using either tangential flow filtration (TFF) or normal-flow filtration (NFF). In this comparison, TFF and NFF were evaluated as preconcentration steps before OMV recovery by ultracentrifugation. The resulting OMV preparations were analyzed using complementary structural and quantitative readouts. **(A)** Representative transmission electron microscopy (TEM) images of OMVs obtained after TFF- or NFF-based preconcentration followed by ultracentrifugation. Scale bars represent 200 nm. **(B)** SDS-PAGE analysis of OMV protein profiles obtained from TFF- and NFF-derived preparations. Each lane was loaded with 30 μg of OMVs, quantified based on total protein content. **(C)** OMV-fraction protein content normalized to viable bacterial density (CFU/mL). **(D)** FM4-64 lipid-associated fluorescence normalized to CFU/mL. Fluorescence values are expressed as arbitrary fluorescence units (AFU). **(E)** Particle number determined by nanoparticle tracking analysis and normalized to CFU/mL. Data are presented as mean ± SEM from at least three independent biological replicates. No statistically significant differences were observed between TFF- and NFF-derived preparations under the tested conditions.

TFF- and NFF-derived preparations showed similar vesicle-like morphology by TEM and broadly similar SDS-PAGE protein profiles (Figures 5A,B). No statistically detectable differences were observed in protein-associated recovery, FM4-64 fluorescence, or NTA-detected particle recovery normalized to viable bacterial density (Figures 5C–E). Mean and modal particle diameters differed numerically, but particles from both conditions remained within the nanometric range measured for the preparations (Supplementary Table 1). Protein-to-particle and lipid-to-particle ratios also showed no statistically detectable differences (Supplementary Figures 3A,B).

Together, these results indicate that TFF can serve as an alternative to NFF as a preconcentration step before ultracentrifugation-based OMV recovery without detectably compromising OMV morphology, apparent recovery, size distribution, global protein profile, or bulk per-particle protein/lipid-associated content. Thus, while this comparison does not by itself establish a fully ultracentrifugation-free workflow, it supports TFF as a suitable downstream platform for subsequent integration with diafiltration and lyophilization.

### 3.4 Storage retains bulk protein- and lipid-associated signals while altering TEM-derived diameter distributions and zeta potential

We next evaluated whether storage condition and duration affected OMV morphology, TEM-derived diameter distributions, bulk biochemical signals, and zeta potential. For this purpose, OMVs prepared under the selected low-agitation condition were stored at 4, −20, −80 °C, or in lyophilized form for up to 120 days. Freshly obtained OMVs were included as the day 0 reference condition. This design allowed us to evaluate whether commonly used preservation conditions maintained vesicle morphology and bulk composition, or instead promoted storage-dependent changes in vesicle organization and physicochemical properties.

**FIGURE 6:**
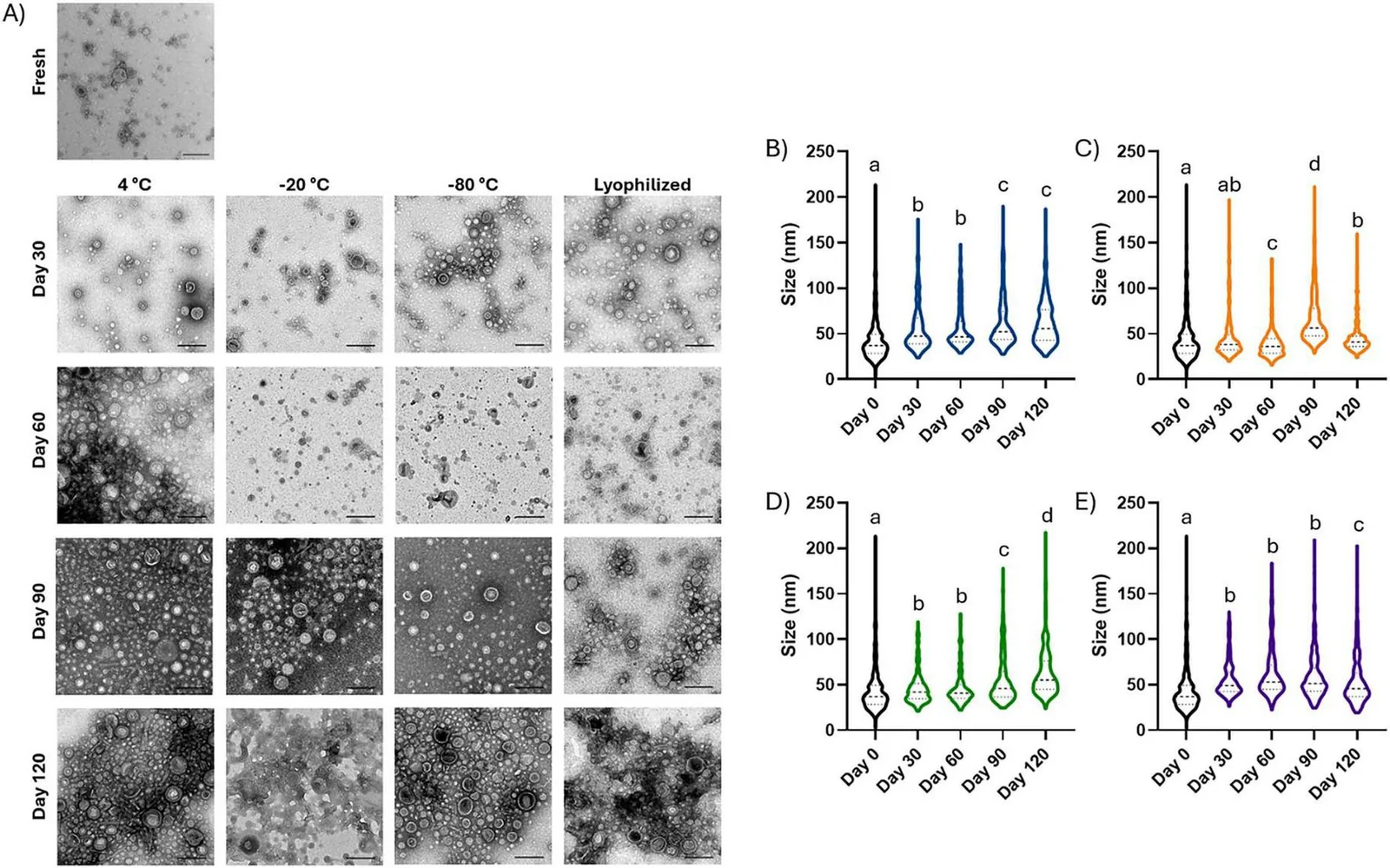
Storage conditions preserve recognizable *S*. Typhi OMV morphology but alter vesicle size distribution over time. **(A)** Representative transmission electron microscopy (TEM) images of *S.* Typhi OMVs stored for up to 120 days under different preservation conditions. Freshly obtained OMVs were analyzed as the Day 0 reference and compared with OMVs stored at 4, −20, −80 °C, or in lyophilized form after reconstitution at days 30, 60, 90, and 120. Images were used to assess vesicle morphology, background heterogeneity, and storage-associated changes in vesicle organization, including clustering or accumulation of vesicle-like structures. Scale bars represent 200 nm. **(B–E)** Violin plots showing vesicle diameter distributions measured from TEM images at each storage time point under the indicated conditions: **(B)** 4 °C, **(C)** −20 °C, **(D)** −80 °C, and **(E)** lyophilized. The dashed line indicates the median vesicle diameter. Data were obtained from three independent biological replicates, with three TEM images analyzed per replicate and at least 100 OMVs measured per replicate. Statistical comparisons among storage time points within each preservation condition were performed using Kruskal–Wallis test followed by Dunn’s multiple-comparison test. Different lowercase letters indicate statistically significant differences; groups sharing at least one letter are not significantly different.

Vesicle-like structures remained recognizable under all preservation conditions for up to 120 days, although later time points showed increased clustering and background heterogeneity, including after reconstitution of lyophilized samples (Figure 6A). TEM-derived diameter distributions shifted over time under all storage conditions, but remained within the measured nanometric range (Figures 6B–E).

To quantify these storage-associated changes, vesicle size distributions were measured from TEM images at each time point. Compared with freshly obtained OMVs, all storage conditions showed significant shifts in size distribution over time (Figures 6B–E). OMVs stored at 4 °C displayed an early change in size distribution beginning at Day 30, which persisted at later time points (Figure 6B). Samples stored at −20 °C also showed time-dependent changes, although the pattern was less uniform across the storage period (Figure 6C). OMVs stored at −80 °C exhibited a more progressive shift across later time points (Figure 6D). Lyophilized OMVs likewise showed significant changes after storage and reconstitution, although the measured vesicle populations remained within the expected nanometric range for bacterial OMVs (Figure 6E). Together, these results indicate that prolonged storage preserves recognizable vesicle morphology but modifies vesicle size distribution and population organization.

**FIGURE 7:**
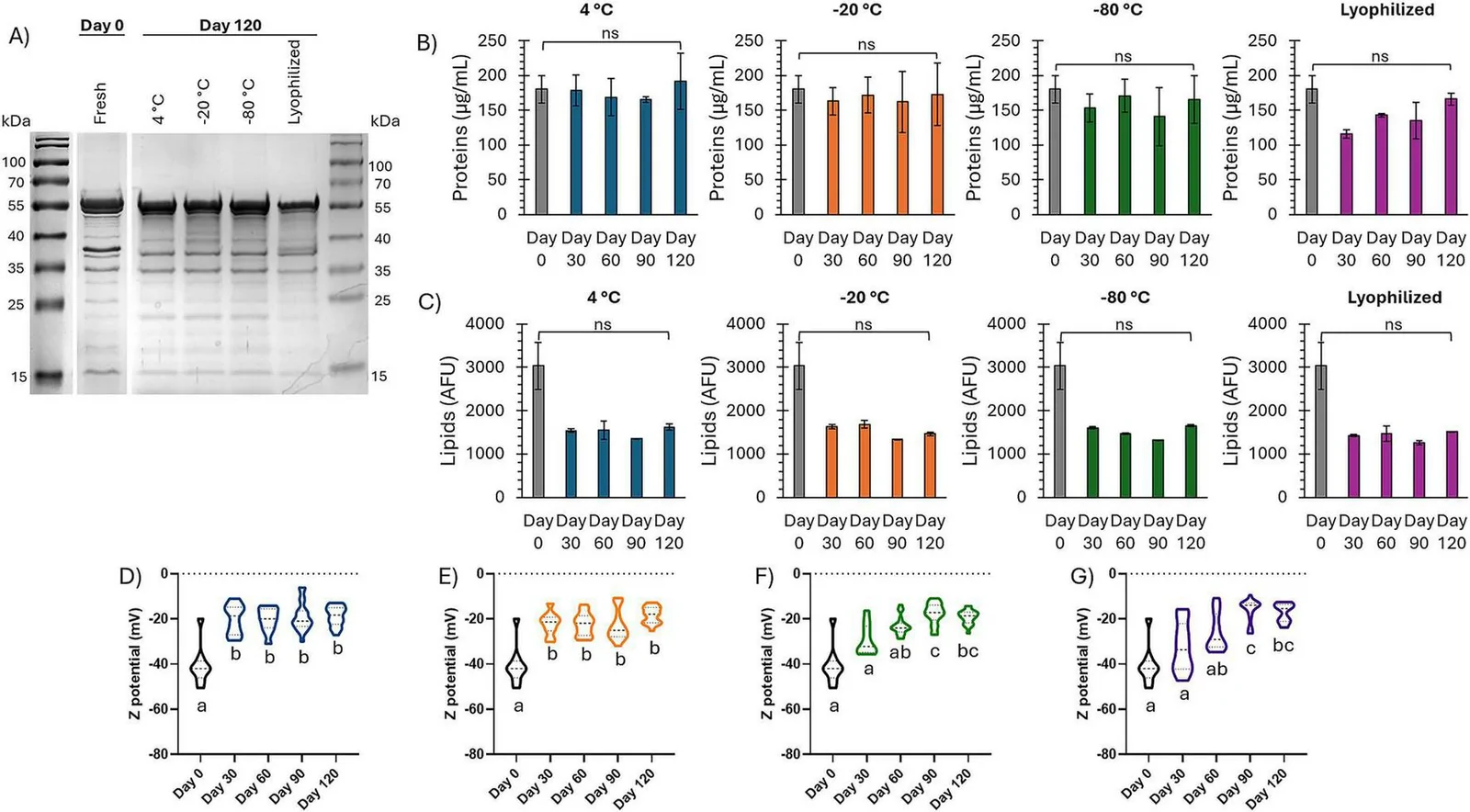
Storage preserves bulk protein and lipid-associated signals but alters *S.* Typhi OMV surface charge over time. **(A)** SDS-PAGE analysis of *S.* Typhi OMVs stored for 120 days under different preservation conditions. Freshly obtained OMVs were included as the day 0 reference and compared with OMVs stored at 4, −20, −80 °C, or in lyophilized form after reconstitution at Day 120. Each lane was loaded with 30 μg of OMVs, quantified based on total protein content, to compare the overall protein banding pattern after storage. **(B)** Total protein content of OMV preparations stored under the indicated conditions for 30, 60, 90, and 120 days, compared with freshly obtained OMVs at day 0. **(C)** Lipid-associated signal measured using FM4-64 fluorescence in OMVs stored under the same conditions and time points. Fluorescence values are expressed as arbitrary fluorescence units (AFU). **(D–G)** Zeta potential distributions of OMVs stored under different conditions: **(D)** 4 °C, **(E)** −20 °C, **(F)** −80 °C, and (G) lyophilized after reconstitution. The dashed line indicates the median zeta potential. Data are presented as mean ± SEM for bulk protein and lipid-associated measurements. For zeta potential violin plots, individual measurements are shown to visualize distribution patterns, with statistical comparisons performed using replicate-level summary values from three independent biological replicates. Statistical comparisons among storage time points within each preservation condition were performed using Kruskal–Wallis test followed by Dunn’s multiple-comparison test (*p* < 0.05). Different lowercase letters indicate statistically significant differences; groups sharing at least one letter are not significantly different.

SDS-PAGE showed no major qualitative loss of dominant bands after 120 days of storage (Figure 7A). Total-protein and FM4-64-associated signals also showed no consistent time-dependent decline across the tested conditions (Figures 7B,C). These bulk measurements do not exclude selective changes in individual proteins, lipids, cargo components, or non-vesicular material. In contrast to the relative preservation of bulk protein and lipid-associated signals, zeta potential analysis revealed marked storage-dependent changes in OMV surface charge (Figures 7D–G). Freshly obtained OMVs showed a more negative zeta potential, whereas stored OMVs generally shifted toward less negative values over time. The magnitude and kinetics of this shift differed among preservation conditions: OMVs stored at 4 and −20 °C showed early changes that remained relatively stable thereafter, whereas −80 °C storage produced a more gradual change across the storage period. Lyophilized OMVs also exhibited altered zeta potential after reconstitution. This reduced magnitude of surface charge is consistent with lower electrostatic stabilization and may have contributed to the increased clustering observed by TEM.

Thus, storage retained recognizable vesicle-like morphology and bulk protein- and lipid-associated signals, but altered TEM-derived diameter distributions, clustering, and surface charge. Preservation of bulk composition therefore did not imply maintenance of an unchanged particle-size distribution, clustering state, or surface charge.

### 3.5 Stored and lyophilized OMV preparations retain detectable cargo-associated activity and epithelial cell-associated fluorescence

Because storage preserved bulk OMV composition but altered vesicle size distribution and surface charge, we next determined whether stored preparations retained detectable cargo-associated activity and the capacity to generate epithelial cell-associated and trypan blue-resistant fluorescence.

**FIGURE 8:**
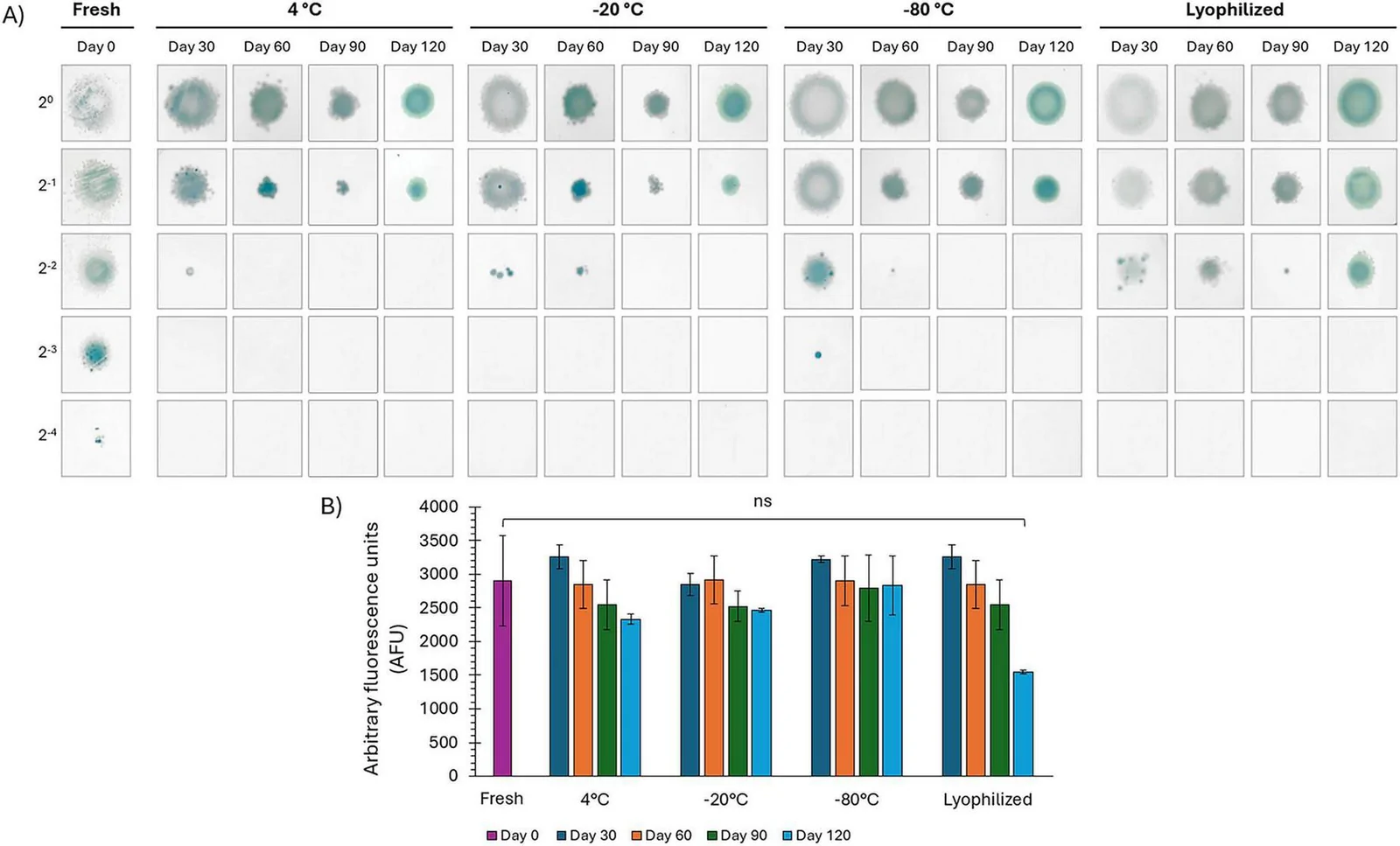
Stored and lyophilized *S*. Typhi OMVs retain detectable cargo-associated enzymatic activity and fluorescent reporter signal. **(A)** β-lactamase activity assay performed using OMVs derived from a *S*. Typhi strain carrying a single chromosomal copy of β-lactamase. OMV preparations were stored at 4, −20, −80 °C, or in lyophilized form for the indicated time points (days 30, 60, 90, and 120). After storage, OMVs were serially diluted and spotted onto LB agar plates containing ampicillin and X-gal in the presence of a β-lactam-sensitive, *lacZ*-positive reporter strain. Detectable β-lactamase activity was visualized as blue reporter signal at the site of OMV deposition, indicating retention of enzymatically active cargo after storage and, for lyophilized samples, reconstitution. **(B)** Quantification of mCherry fluorescence in OMVs derived from a *S*. Typhi strain expressing OMV-targeted mCherry. Fluorescence was measured in OMV preparations stored under the same preservation conditions and time points using equal volumes of OMV preparations reconstituted to the same final volume after each preservation condition and is reported as arbitrary fluorescence units (AFU). Values were not normalized to particle concentration or total protein and therefore represent preparation-level reporter fluorescence. Consequently, this analysis does not distinguish loss of reporter fluorescence within retained OMVs from a reduction in the abundance or recoverability of particles during storage. Data are presented as mean ± SEM from three independent biological replicates. Statistical significance was assessed using one-way ANOVA followed by Tukey’s multiple-comparison test. ns, not significant.

We first assessed enzymatic cargo preservation using OMVs derived from a *S.* Typhi strain carrying a single chromosomal copy of β-lactamase. Freshly obtained OMVs produced clear β-lactamase-positive signals in the reporter assay, confirming the presence of functionally active enzyme associated with the vesicle preparation (Figure 8A). β-Lactamase activity remained detectable after storage under all tested conditions, including in lyophilized preparations after reconstitution (Figure 8A). The apparent signal and detectable dilution range varied among conditions and time points; however, because the assay was semiquantitative, these differences should not be interpreted as precise estimates of retained enzymatic activity. OMVs stored at 4 and −20 °C showed a more evident reduction in detectable activity during prolonged storage, whereas OMVs stored at −80 °C retained a broader activity signal across the storage period. Importantly, lyophilized OMVs also retained detectable β-lactamase activity after reconstitution throughout the evaluated time course, indicating that enzymatic cargo remained functionally detectable after freeze-drying and storage.

To examine whether preservation extended to a fluorescent protein cargo, we next quantified mCherry-associated fluorescence at the level of the recovered OMV preparation. Measurements were performed using equal sample volumes derived from equivalent starting culture volumes and, when applicable, reconstituted to the same final volume. Because fluorescence was not normalized to particle number, the assay reflects the total detectable reporter-associated signal per unit volume of preparation rather than fluorescence per particle. OMVs derived from the mCherry-expressing strain retained measurable fluorescence under all storage conditions throughout the 120-day period (Figure 8B). However, fluorescence preservation was not identical across conditions. OMVs stored at 4 and −20 °C showed a gradual reduction in fluorescence relative to freshly obtained vesicles, whereas OMVs stored at −80 °C maintained fluorescence values closer to early time points. Lyophilized OMVs also retained detectable mCherry fluorescence after reconstitution, although a more pronounced decrease was observed at day 120 compared with earlier time points. Thus, both enzymatic activity and fluorescent reporter signal remained detectable after storage, although their preparation-level retention was condition- and cargo-dependent. The observed reduction in mCherry fluorescence cannot, however, be assigned specifically to degradation or loss of fluorescent competence of mCherry within otherwise intact vesicles, because a reduction in recoverable particle number or other storage-associated effects on signal detection may also have contributed.

**FIGURE 9:**
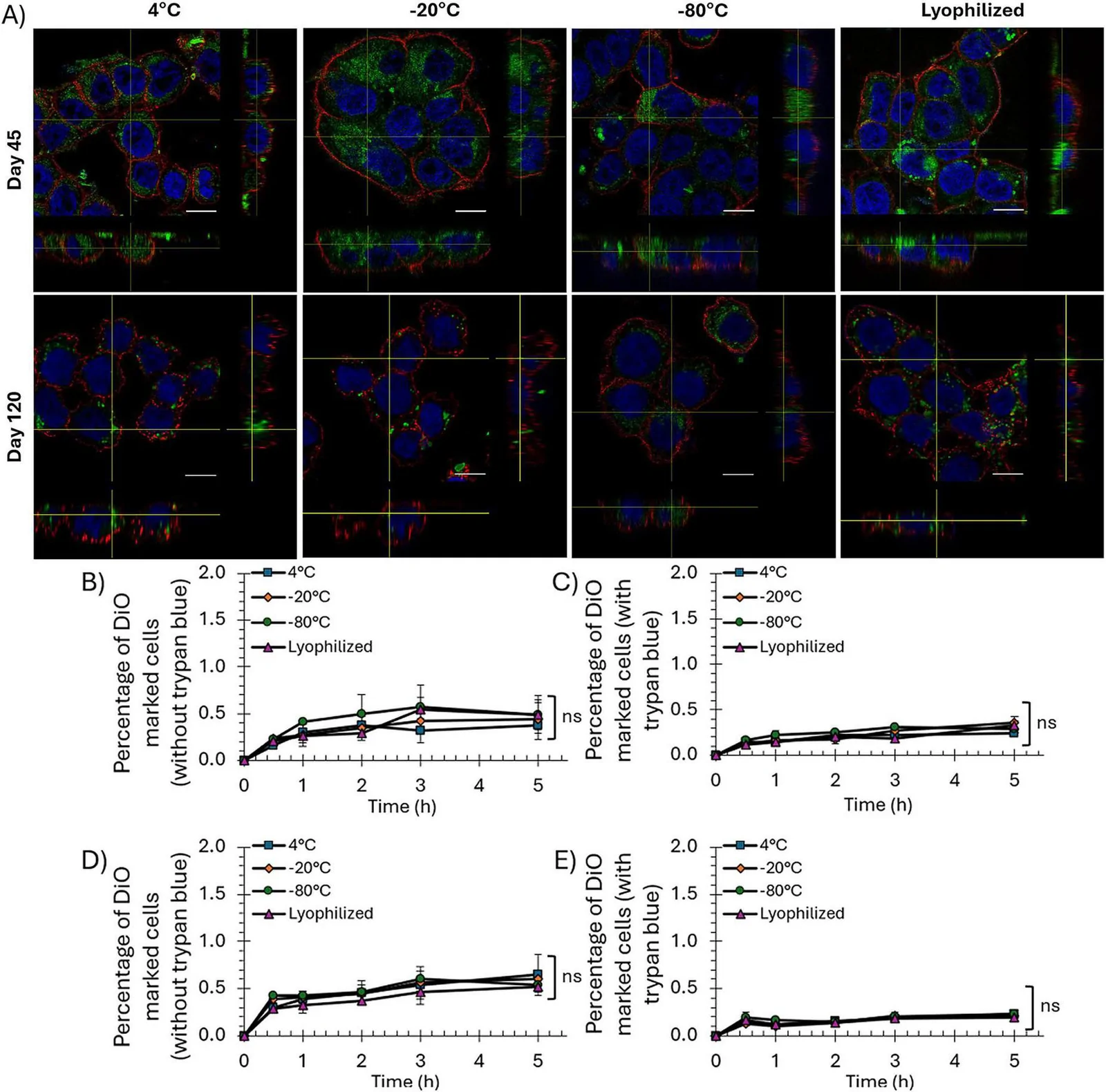
Stored and lyophilized *S*. Typhi OMVs retain epithelial cell association and trypan blue-resistant fluorescence after prolonged storage. **(A)** Representative confocal microscopy images of HT-29 epithelial cells incubated with 50 μg/mL DiO-labeled *S.* Typhi OMVs stored at 4, −20, −80 °C, or in lyophilized form for 45 or 120 days. Lyophilized OMVs were reconstituted before cell exposure. OMV-associated DiO fluorescence is shown in green, nuclei are shown in blue, and the plasma membrane is shown in red. Images were used to qualitatively assess whether stored OMVs retained the capacity to associate with epithelial cells after prolonged preservation. **(B–E)** Flow cytometry analysis of HT-29 cells exposed to 100 μg/mL DiO-labeled OMVs stored under the indicated conditions (gating strategy shown in Supplementary Figure 5). Cells were incubated with OMVs stored for 45 days **(B,C)** or 120 days **(D,E)**, and fluorescence was measured over time. Total cell-associated DiO fluorescence, measured in the absence of trypan blue quenching, is shown in panels (B) and **(D)**. Trypan blue-resistant fluorescence, measured after quenching of extracellular DiO signal, is shown in panels **(C,E)** and was used as an uptake-associated readout enriched for internalized OMVs. Results are expressed as the percentage of DiO-positive HT-29 cells at each time point. Data are presented as mean ± SEM from three independent biological replicates. Statistical significance was determined using two-way ANOVA followed by Tukey’s multiple-comparison test.

We then evaluated whether the preservation of cargo-associated activity was accompanied by maintenance of OMV interaction with epithelial cells. HT-29 cells were incubated with DiO-labeled OMVs stored under the different preservation conditions, and OMV–cell interaction was assessed by confocal microscopy and flow cytometry. Confocal microscopy showed detectable DiO-associated signal in close association with HT-29 cells after exposure to OMVs stored for either 45 or 120 days (Figure 9A). This signal was observed across all storage conditions, including lyophilized OMVs after reconstitution, indicating that prolonged storage did not abolish OMV–cell association. Nevertheless, qualitative differences in fluorescence intensity and spatial distribution were observed among conditions, suggesting that preservation may affect the magnitude or organization of vesicle–cell interaction without eliminating it.

To quantify total cell-associated and trypan blue-resistant fluorescence, HT-29 cells were analyzed by flow cytometry in the absence or presence of extracellular fluorescence quenching. In the absence of trypan blue, OMVs stored for 45 days showed a time-dependent increase in DiO-positive HT-29 cells, reaching a plateau after approximately 2–3 h of incubation (Figure 9B). Measurements performed in the presence of trypan blue retained a substantial fluorescence signal, consistent with internalized or otherwise quenching-resistant OMV-associated fluorescence (Figure 9C). Similar uptake-associated kinetic profiles were observed for OMVs stored for 120 days (Figures 9D,E). Across both storage periods, all conditions retained the capacity to generate cell-associated and trypan blue-resistant fluorescence, supporting retention of cell-associated and quenching-resistant OMV-associated fluorescence after storage.

Collectively, stored preparations retained detectable β-lactamase activity, preparation-level mCherry fluorescence, and epithelial cell-associated fluorescence, including after lyophilization and reconstitution. However, the magnitude of retention depended on the measured cargo and preservation condition, and the observed physicochemical changes indicate that lyophilization did not preserve all preparation attributes unchanged.

### 3.6 Diafiltration performed after TFF preconcentration retains key measured OMV attributes in an ultracentrifugation-free workflow

We finally compared four workflows that shared TFF as the initial preconcentration step but differed in the subsequent processing branch and lyophilization status. In the reference workflows, TFF preconcentration was followed by ultracentrifugation, either without or with subsequent lyophilization. In the ultracentrifugation-free workflows, the retentate remained in the same TFF system and was subsequently diafiltered using either NaCl or sucrose solution, followed by lyophilization. Thus, after the common TFF preconcentration step, ultracentrifugation was replaced by diafiltration performed in the same TFF system.

**FIGURE 10:**
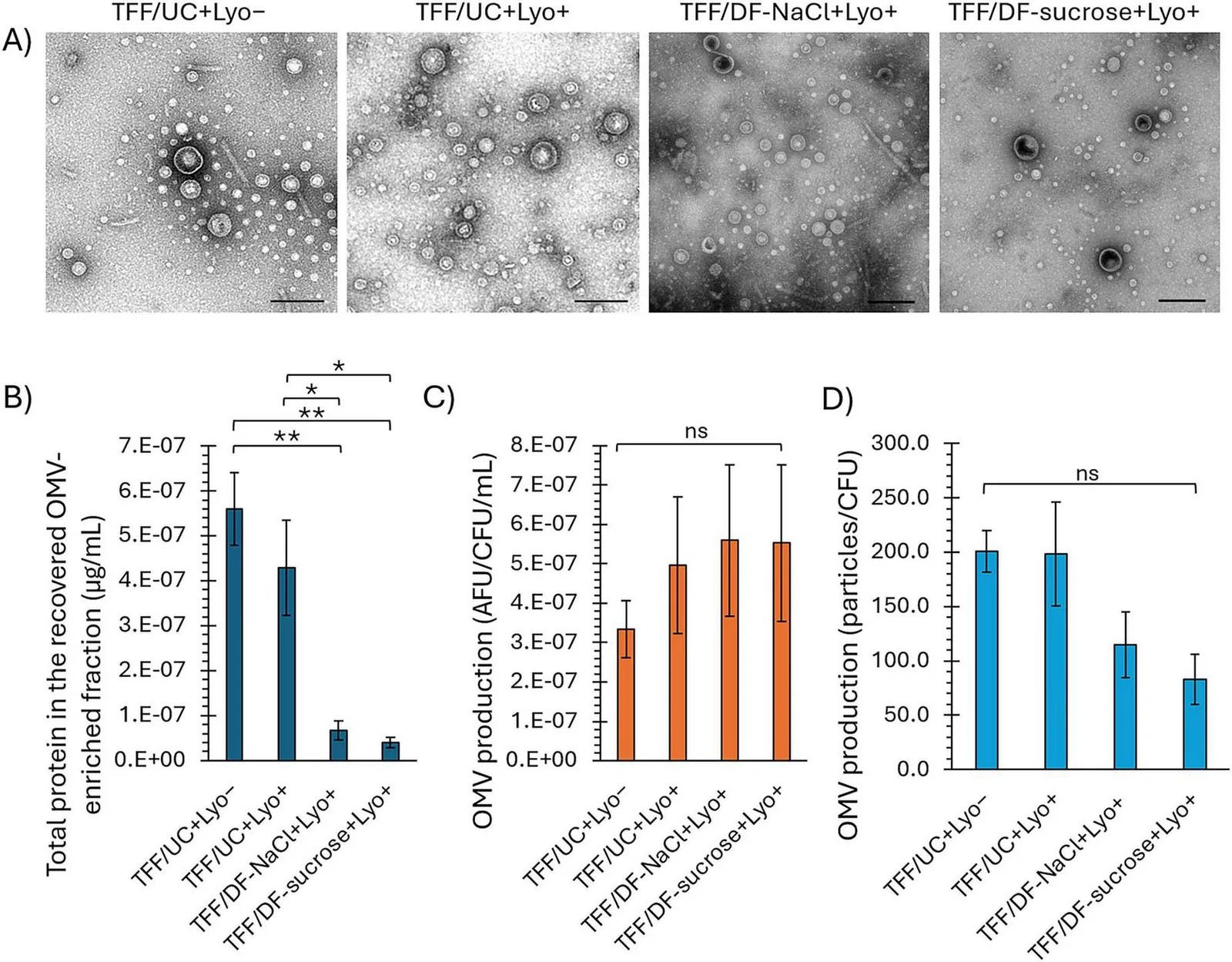
Diafiltration replaces ultracentrifugation within a TFF-based OMV workflow while preserving recognizable vesicular morphology and key recovery readouts. **(A)** Representative transmission electron microscopy (TEM) images of *S.* Typhi OMVs obtained using four processing workflows sharing TFF as the initial preconcentration step. In the reference workflows, TFF was followed by ultracentrifugation without or with subsequent lyophilization (TFF/UC+Lyo− and TFF/UC+Lyo+, respectively). In the ultracentrifugation-free workflows, TFF preconcentration was followed by diafiltration performed in the same TFF system using either NaCl or sucrose as the exchange solution and subsequent lyophilization (TFF/DF-NaCl+Lyo+ and TFF/DF-sucrose+Lyo+, respectively). Images were used to evaluate vesicle morphology and gross structural preservation across processing conditions. Scale bars represent 200 nm. **(B)** Total protein content in the recovered OMV-enriched fraction normalized to viable bacterial density (CFU). **(C)** OMV-associated lipid fluorescence measured using FM4-64 and normalized to viable bacterial density (CFU). **(D)** Particle counts determined by nanoparticle tracking analysis (NTA) and normalized to viable bacterial density (CFU). Data are presented as mean ± SEM from three independent biological replicates. Statistical significance is indicated by asterisks (*p < 0.05; **p < 0.01); non-significant comparisons are indicated as ns where applicable.

Four processing workflows were compared, all of which used TFF as the initial preconcentration step. In the first reference workflow, TFF-preconcentrated OMVs were subsequently recovered by ultracentrifugation and analyzed without lyophilization (TFF/UC+Lyo−). A second reference condition used the same TFF/ultracentrifugation sequence followed by lyophilization (TFF/UC+Lyo+), allowing the effect of lyophilization to be assessed within the ultracentrifugation branch. In the two ultracentrifugation-free workflows, the TFF retentates were diafiltered using either NaCl or sucrose as the exchange solution and subsequently lyophilized; these conditions were designated TFF/DF-NaCl+Lyo+ and TFF/DF-sucrose+Lyo+, respectively. All final OMV preparations were resuspended in 1 mL of PBS, thereby standardizing the final resuspension solvent and volume across workflows. Comparison of TFF/UC+Lyo− and TFF/UC+Lyo+ allowed the effect of lyophilization to be assessed within the ultracentrifugation-based branch, whereas the TFF/DF conditions were interpreted as integrated diafiltration–lyophilization workflows. Thus, TFF was common to all four workflows, whereas the distinguishing post-concentration operation was ultracentrifugation or diafiltration. TEM showed recognizable vesicle-like structures in all workflows, with no gross collapse or extensive fragmentation after reconstitution of the ultracentrifugation-free preparations (Figure 10A).

We then compared apparent OMV-associated recovery using complementary readouts. When normalized to viable bacterial density, both ultracentrifugation-derived preparations, TFF/UC+Lyo− and TFF/UC+Lyo+, showed higher total protein content in the recovered OMV-enriched fraction than the ultracentrifugation-free conditions, TFF/DF-NaCl+Lyo+ and TFF/DF-sucrose+Lyo+, whereas no significant difference was observed between the two TFF/UC-derived workflows (Figure 10B). In contrast, lipid-associated fluorescence and particle counts did not differ significantly among workflows (Figures 10C,D). Because lipid- and particle-associated readouts were not significantly reduced and protein-to-particle ratios remained comparable among workflows (Supplementary Figure 4A), the lower total-protein signal may reflect reduced carryover of soluble or other non-vesicular proteins during diafiltration; however, proportional particle loss or other workflow-dependent effects cannot be excluded.

NTA further showed that all workflows yielded particles within the expected nanometric range for bacterial OMVs (Table 4). Although numerical differences in mean and modal diameter were observed among conditions, particle size distributions remained compatible with OMV preparations across both conventional and ultracentrifugation-free workflows. Particle concentrations were numerically lower in the ultracentrifugation-free preparations, but no statistically significant difference among workflows was detected (Table 4). Given the number of biological replicates, these results do not establish formal equivalence in particle recovery. Consistent with this observation, protein- and lipid-associated signals normalized to particle number did not differ significantly among workflows (Supplementary Figures 4A,B), suggesting that the recovered particles showed no statistically detectable differences in bulk protein-to-particle or lipid-to-particle ratios.

**TABLE 4:** Nanoparticle tracking analysis of *S.* Typhi OMV preparations obtained using TFF-based workflows followed by ultracentrifugation or diafiltration.

Condition	Post-TFF operation	Diafiltration exchange solution	Lyophilization	Mean size (nm)	Mode (nm)	Concentration (particles/mL)
TFF/UC+Lyo−	Ultracentrifugation	−	No	135.5 ± 2.2	112.7 ± 3.0	3.92 × 10^11^ ± 1.93 × 10^10^
TFF/UC+Lyo+	Ultracentrifugation	−	Yes	122.7 ± 2.8	100.1 ± 7.0	5.73 × 10^11^ ± 7.45 × 10^9^
TFF/DF-NaCl+Lyo+	Diafiltration	NaCl	Yes	136.4 ± 3.6	107.5 ± 11.4	2.14 × 10^11^ ± 1.68 × 10^10^
TFF/DF-sucrose+Lyo+	Diafiltration	Sucrose	Yes	141.0 ± 4.8	116.3 ± 5.4	1.57 × 10^11^ ± 5.62 × 10^9^

OMVs were prepared from *S.* Typhi cultures grown under the selected low-agitation condition and processed using four workflows sharing TFF as the initial preconcentration step. Reference workflows subsequently used ultracentrifugation without or with lyophilization (TFF/UC+Lyo− and TFF/UC+Lyo+, respectively). In the ultracentrifugation-free workflows, the ultracentrifugation step was replaced by diafiltration using either NaCl or sucrose, followed by lyophilization (TFF/DF-NaCl+Lyo+ and TFF/DF-sucrose+Lyo+, respectively). Particle size distribution and concentration were determined by nanoparticle tracking analysis (NTA). Mean particle diameter, modal diameter, and particle concentration are reported for each workflow. Data are presented as mean ± SEM from three independent biological replicates.

**FIGURE 11:**
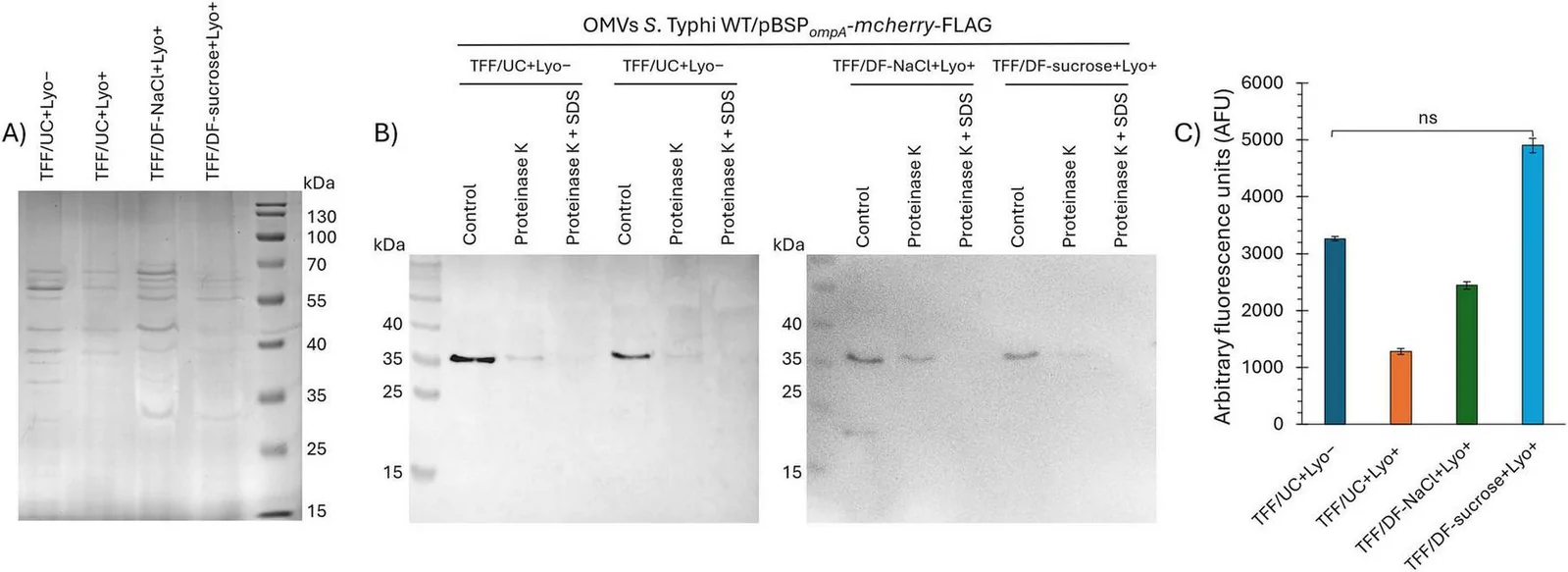
Preservation of OMV-associated protein profile, membrane-protected mCherry-FLAG cargo, and fluorescent reporter signal after replacing ultracentrifugation with diafiltration in a TFF-based workflow. **(A)** SDS-PAGE analysis of *S.* Typhi OMVs obtained using four workflows sharing TFF as the initial preconcentration step: TFF followed by ultracentrifugation without or with lyophilization (TFF/UC+Lyo− and TFF/UC+Lyo+), or TFF preconcentration followed by diafiltration of the retained material in the same TFF system using NaCl or sucrose as the exchange solution, and subsequent lyophilization (TFF/DF-NaCl+Lyo+ and TFF/DF-sucrose+Lyo+, respectively). Protein profiles were compared to assess preservation of the global OMV-associated protein pattern across processing conditions. **(B)** Proteinase K protection assay performed on OMVs expressing OmpA signal peptide–targeted mCherry fused to a C-terminal FLAG tag (SP*_*ompA*_*–mCherry-FLAG), a reporter previously shown to accumulate in *S*. Typhi OMVs (Fuentes et al., 2025). OMVs obtained from each workflow were incubated with proteinase K in the absence or presence of SDS, and mCherry-FLAG was detected by Western blot using an anti-FLAG antibody. Protection of the mCherry-FLAG signal from proteinase K digestion in the absence of SDS, together with loss or reduction of the signal after detergent-mediated membrane disruption, was used as a readout of membrane-dependent cargo protection and vesicle integrity. **(C)** Quantification of mCherry fluorescence in OMVs expressing OmpA signal peptide–targeted mCherry. Fluorescence is reported as arbitrary fluorescence units (AFU) and was used to evaluate preservation of detectable fluorescent cargo-associated signal following each processing workflow. Measurements were performed using equal volumes of OMV preparations reconstituted to the same final volume after each processing condition. Data are presented as mean ± SEM from three independent biological replicates. Statistical significance was assessed using one-way ANOVA followed by Tukey’s multiple-comparison test. ns, not significant.

We next examined whether the ultracentrifugation-free workflows preserved the OMV protein profile and cargo integrity. SDS-PAGE analysis revealed broadly similar protein banding patterns across conventional TFF/UC+Lyo− and TFF/UC+Lyo+ preparations and ultracentrifugation-free TFF/DF-NaCl+Lyo+ and TFF/DF-sucrose+Lyo+ preparations, with no major qualitative loss of dominant protein bands detected under the conditions tested (Figure 11A). To evaluate membrane-dependent cargo protection, we used a previously validated OMV-targeted fluorescent reporter based on the *S.* Typhi OmpA signal peptide fused to mCherry and a C-terminal FLAG tag (SP*_*ompA*_*–mCherry-FLAG). This Sec-targeted construct was previously shown to promote mCherry-FLAG accumulation within *S.* Typhi OMVs, enabling its detection by anti-FLAG Western blot and its use as a reporter of heterologous protein loading into vesicles (Fuentes et al., 2025). In the proteinase K protection assay, mCherry-FLAG remained detectable in the absence of detergent but was reduced or lost after SDS-mediated membrane disruption, indicating retention of membrane-dependent protease protection after ultracentrifugation-free processing (Figure 11B).

Finally, we assessed whether fluorescent reporter signal was preserved following ultracentrifugation-free processing. Both diafiltration-based workflows retained detectable mCherry fluorescence after reconstitution, with no statistically detectable difference between the NaCl- and sucrose-based conditions (Figure 11C).

**FIGURE 12:**
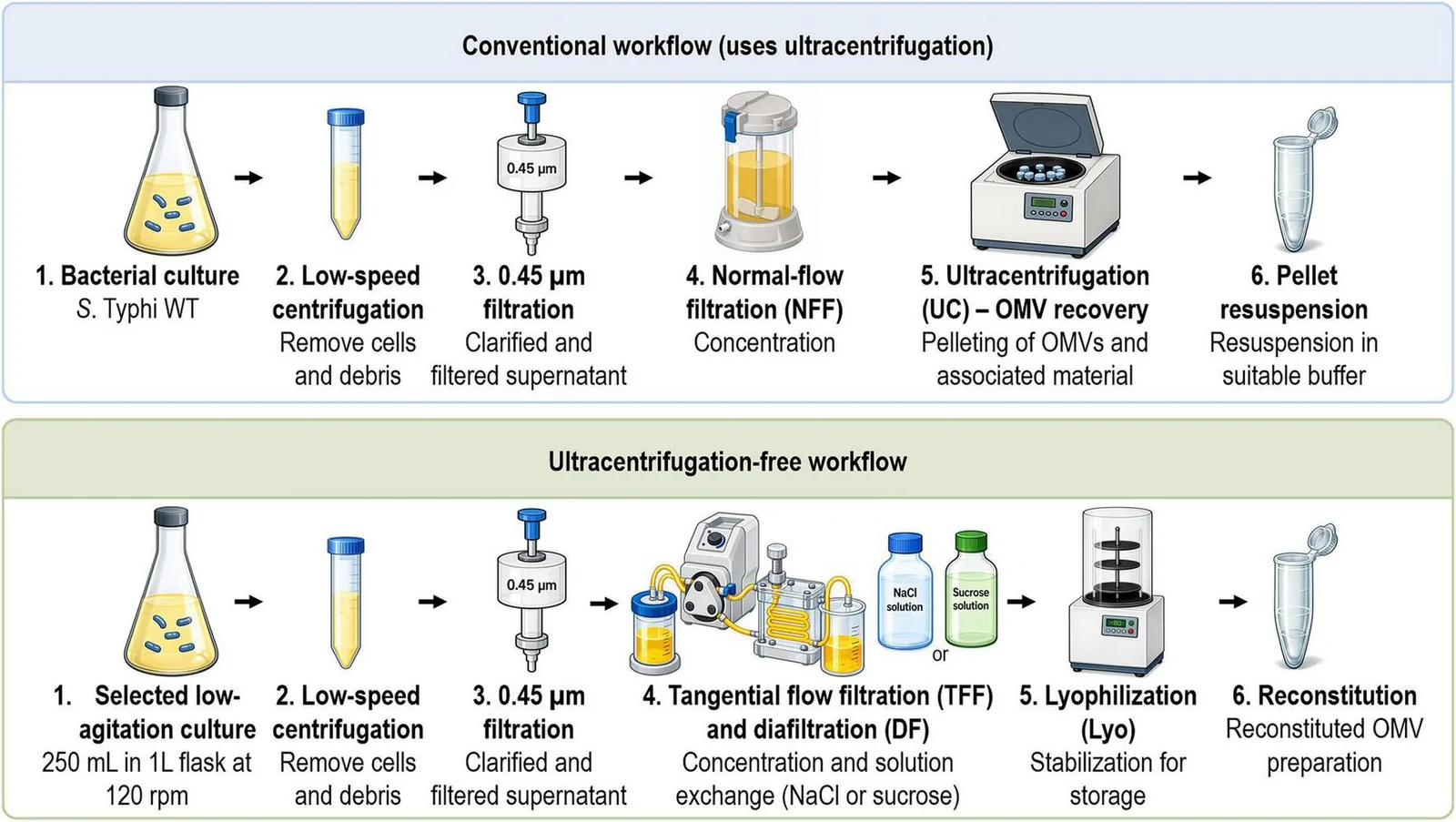
Schematic comparison of a traditional ultracentrifugation-based workflow and the ultracentrifugation-free workflow developed in this study. The upper branch illustrates a representative traditional OMV-processing workflow consisting of bacterial culture, low-speed centrifugation, 0.45 μm filtration, normal-flow filtration (NFF)-based concentration, ultracentrifugation-based OMV recovery, and pellet resuspension. This branch is presented as a conceptual reference for conventional OMV processing and does not correspond to the matched experimental comparator used in Figures 10, 11, in which TFF was used as the initial preconcentration step in all evaluated workflows. The lower branch represents the ultracentrifugation-free workflow developed in this study, using the selected low-agitation culture condition (250 mL culture volume in 1 L Erlenmeyer flasks at 120 rpm) followed by low-speed centrifugation, 0.45 μm filtration, TFF-based preconcentration, diafiltration performed in the same TFF system using either NaCl or sucrose as the exchange solution, lyophilization, and reconstitution. The schematic highlights the ultracentrifugation-free branch, in which TFF-based preconcentration is followed by diafiltration in the same TFF system, rather than ultracentrifugation, and subsequently by lyophilization and reconstitution. UC, ultracentrifugation; NFF, normal-flow filtration; TFF, tangential flow filtration; DF, diafiltration; Lyo, lyophilization.

Overall, the TFF/DF/lyophilization workflows, in which diafiltration replaced the ultracentrifugation step after the common TFF preconcentration stage, retained recognizable vesicular morphology, nanometric particles, dominant SDS-PAGE protein bands, membrane-dependent mCherry-FLAG protection, and detectable reporter fluorescence. Although total-protein-associated recovery was lower than in TFF/UC-derived preparations, lipid- and particle-associated readouts showed no statistically detectable reductions. Taken together, these results support the feasibility of the evaluated ultracentrifugation-free workflow, in which ultracentrifugation was replaced by diafiltration after TFF preconcentration and followed by lyophilization, while identifying compositional recovery and colloidal stability as priorities for further optimization. A schematic comparison of the ultracentrifugation-based and ultracentrifugation-free workflows is provided in Figure 12.

## 4 Discussion

Outer membrane vesicles are increasingly explored as biologically active bacterial nanoparticles for mechanistic microbiology, vaccine development, antigen and protein delivery, and bacterial nanobiotechnology, yet their broader implementation as nanoscale biological platforms remains limited by the difficulty of producing preparations that are reproducible, sufficiently defined, scalable, and stable (Sartorio et al., 2021; Juodeikis and Carding, 2022). In this study, we addressed this limitation by treating *S.* Typhi OMV production as an integrated process-development problem in which process performance and OMV quality attributes were evaluated jointly rather than through particle recovery alone. Our results show that upstream culture conditions, preconcentration strategy, storage format, and final downstream unit-operation sequence collectively influence process-performance readouts and structural, biochemical, physicochemical, cargo-associated, and functional OMV quality attributes. The main contribution of this work is the development of a research-grade TFF-, diafiltration-, and lyophilization-based workflow that reduces dependence on ultracentrifugation while preserving key measured OMV attributes, including recognizable vesicular morphology, nanoscale particle size, global SDS-PAGE protein profile, membrane-protected mCherry-FLAG cargo, and detectable reporter fluorescence. Importantly, these OMV quality attributes were preserved even though not all process-performance readouts were maximized, reinforcing the need to assess OMV quality independently of apparent recovery alone.

Our culture-condition experiments demonstrate that the upstream stage directly shapes both the apparent recovery and the quality attributes of the material entering the downstream workflow. This is consistent with previous studies showing that bacterial physiological state and culture-related parameters can influence vesicle yield, physicochemical properties, cargo composition, and cell-association activity (Schwechheimer and Kuehn, 2015; Orench-Rivera and Kuehn, 2016). In our system, increased agitation enhanced apparent OMV-associated recovery, particularly when assessed by protein content and particle number, but also increased the co-isolation of filamentous material and flagellin signal in WT preparations. Thus, higher process-performance readouts were not necessarily associated with more favorable OMV quality attributes. By contrast, the 120 rpm/250 mL condition provided a more favorable balance between recovery, reduced flagellin carryover, cleaner vesicle morphology, and preserved epithelial cell-associated and trypan blue-resistant fluorescence in HT-29 cells. These findings indicate that culture conditions should be selected according to contaminant carryover, vesicle morphology, and downstream functional performance, rather than evaluated solely by crude vesicle-associated yield.

The reduction of flagellin carryover is particularly relevant for *Salmonella*-derived OMVs. Flagellin is not an inert contaminant; it is a potent immunostimulatory molecule recognized by Toll-like receptor 5 and has been exploited as an adjuvant in vaccine formulations (Hayashi et al., 2001; Skountzou et al., 2010). Therefore, its co-purification can distort biochemical and functional interpretation, especially in assays involving host-cell responses. Previous studies using flagellin-deficient *S.* Typhimurium OMVs have shown that genetic removal of flagellin can improve antigenic definition and support protective immune responses Liu et al. (2016a) is the study using flagellin-deficient *S*. Typhimurium OMVs, whereas Liu et al. (2016b) concerns truncated-LPS mutants. In the present study, the absence of filamentous structures and anti-flagellin signal in Δ*fliC*-derived preparations supports the assignment of the filamentous material observed in WT preparations to flagellar or flagellin-associated structures. Importantly, our optimized low-agitation condition reduced this carryover without genetically modifying the producer strain, providing a non-genetic upstream strategy to improve the interpretability of *S.* Typhi OMV preparations. Nevertheless, the association between reduced flagellin carryover and increased epithelial cell-associated fluorescence should not be interpreted as proof of direct causality. Other agitation-dependent changes, including vesicle heterogeneity, aggregation state, surface composition, or co-isolated material, may also contribute to this phenotype.

The mechanism by which agitation increased flagellin-associated carryover remains unresolved. Because the material detected by TEM and Western blot was recovered within the OMV-enriched fraction, our data support the co-isolation of flagellin-containing filamentous material, but do not distinguish whether this material results from mechanically detached flagellar fragments, increased flagellin release, altered flagellar assembly, or changes in flagellar expression. Higher agitation can modify oxygen transfer, mixing efficiency, shear exposure, and bacterial physiological state in shake-flask cultures, all of which may influence extracellular vesicle release and the composition of material entering the purification workflow (Duetz et al., 2000; Orench-Rivera and Kuehn, 2016; Gerritzen et al., 2018). Therefore, the higher flagellin signal observed at 180 rpm may result from a combination of mechanical and physiology-dependent effects. Although additional experiments, such as quantitative flagellin analysis in cellular and extracellular fractions or assessment of flagellar gene expression under each agitation condition, would be required to resolve the underlying mechanism, the present data show that low-agitation culture reduces flagellin-associated contaminant carryover and improves the definition of the OMV-enriched preparation within the optimized workflow.

The potential impact of flagellin-containing material on epithelial cell-associated fluorescence should also be interpreted cautiously. Because OMV inputs in the HT-29 assays were normalized by total protein, preparations enriched in non-vesicular flagellin-containing material may contain a lower effective amount of vesicle-associated lipid or vesicle-competent particles per unit of protein. In this scenario, reduced cell-associated or trypan blue-resistant fluorescence may reflect dilution of vesicle-associated material, altered vesicle accessibility, increased aggregation, or steric interference by co-isolated filamentous structures, rather than a direct inhibitory effect of flagellin on OMV uptake. This interpretation is consistent with previous evidence showing that OMV size, composition, and population heterogeneity can influence epithelial cell entry and vesicle-associated cargo delivery (O’Donoghue and Krachler, 2016; Turner et al., 2018). Accordingly, the relationship between flagellin carryover and epithelial interaction is likely to be context-dependent and may vary across bacterial species, vesicle composition, purification workflow, recipient cell type, and normalization strategy. For this reason, the reduced flagellin carryover observed under low-agitation conditions should be viewed primarily as a reduction in contaminant carryover and an improvement in functional interpretability, rather than as a universally predictive determinant of OMV uptake.

The comparison between TFF and NFF supports the use of TFF as a scale-compatible preconcentration platform. When both approaches were used before ultracentrifugation, TFF did not significantly alter OMV-fraction protein recovery, lipid-associated fluorescence, particle yield, size distribution, or bulk per-particle protein/lipid-associated content relative to NFF. This indicates that, under the tested conditions, TFF can serve as an alternative to NFF as a preconcentration step without causing gross loss or detectable alteration of major OMV attributes. The advantage of TFF is therefore not necessarily increased recovery at this stage, but its suitability for process integration: TFF can handle larger volumes, reduce dead-end membrane fouling, and be coupled to diafiltration for solution exchange and formulation-compatible downstream processing (Watson et al., 2021; Torres-Vanegas et al., 2024). This distinction is important because Figure 5 does not establish a fully ultracentrifugation-free workflow by itself; rather, it validates TFF as a suitable platform for subsequent integration with diafiltration and lyophilization.

Storage experiments revealed that preservation of major OMV features can occur alongside substantial changes in particle-size distribution, clustering, and surface charge. Across refrigerated, frozen, and lyophilized conditions, OMVs retained recognizable vesicular morphology and broadly preserved bulk protein and lipid-associated signals, suggesting that storage did not abolish the major structural and biochemical features detected by TEM, SDS-PAGE, protein quantification, and FM4-64 fluorescence. However, these readouts were accompanied by storage-dependent shifts in size distribution, increased clustering or background heterogeneity, and marked changes in zeta potential. This partial dissociation between biochemical preservation and colloidal stability is consistent with extracellular vesicle studies showing that storage can alter particle concentration, size, purity, aggregation, zeta potential, and downstream functionality even when vesicle-associated material remains detectable (Jeyaram and Jay, 2017; Ahmadian et al., 2024). Therefore, OMV stability should not be inferred from protein or lipid retention alone. Applications requiring controlled dispersion, surface charge, reproducible cell interaction, or defined particle populations may be particularly sensitive to storage-induced physicochemical changes.

Lyophilization was compatible with the preservation of several functional readouts, although its effects depended on the cargo and endpoint evaluated. Freeze-drying has been proposed as a strategy to improve extracellular vesicle storage, particularly when stabilizing excipients are included, but its performance must be assessed using multiple structural and functional parameters (Trenkenschuh et al., 2022; Ahmadian et al., 2024). In our system, lyophilized *S*. Typhi OMVs retained detectable β-lactamase activity after reconstitution, indicating that enzymatic cargo can remain functionally recoverable after drying. This agrees with previous evidence showing that directed packaging into bacterial OMVs can protect enzymatic function under stress conditions, including lyophilization (Alves et al., 2016). OMVs carrying the SP*_*ompA*_*–mCherry-FLAG reporter also retained measurable fluorescence after storage and reconstitution, supporting retention of a detectable reporter-associated signal (Fuentes et al., 2025). However, because fluorescence was measured using equal preparation volumes rather than normalized to matched particle counts, the time-dependent reduction in signal cannot be attributed specifically to physical degradation of mCherry within otherwise intact vesicles. General particle loss, changes in recoverable particle abundance, aggregation-related effects on detection, loss of mCherry fluorescent competence without complete protein degradation, or a combination of these processes may have contributed (Gelibter et al., 2022). The preservation of bulk protein and lipid-associated signals argues against extensive nonspecific loss of the total recovered material, but these bulk measurements do not directly establish preservation of particle number or mCherry abundance. Discriminating between particle-level loss and cargo-specific destabilization would require particle concentration measurements at each storage time point, particle-normalized fluorescence, and ideally quantitative analysis of mCherry-FLAG abundance and integrity.

The behavior of β-lactamase activity and mCherry fluorescence differed across conditions and time points, emphasizing that apparent cargo preservation depends on both the protein and the analytical readout employed. Similarly, stored and lyophilized OMVs retained the capacity to generate cell-associated and trypan blue-resistant fluorescence in HT-29 cells, although these assays should be interpreted as uptake-associated fluorescence readouts rather than definitive evidence of preserved OMV internalization dynamics. Future studies should combine immunogold transmission electron microscopy of ultrathin HT-29 cell sections to localize OMV-specific markers within membrane-bounded intracellular compartments with cell-surface stripping followed by subcellular fractionation and detection of the intravesicular mCherry-FLAG reporter; concordant ultrastructural and biochemical evidence would more definitively distinguish true OMV internalization from persistent surface association or membrane-dye transfer (Bitto et al., 2017; Pavkova et al., 2021).

In the ultracentrifugation-free branch, the culture supernatant was first concentrated by TFF, after which the retained material was diafiltered in the same TFF system before lyophilization. This workflow should be interpreted as a process-integration strategy rather than as a simple replacement of one isolation step by another. Conventional OMV isolation procedures differ in recovery, purity, vesicle size distribution, and co-isolated material, making the workflow itself a determinant of the final preparation (Torres-Vanegas et al., 2024). In our final comparison, the TFF/DF-NaCl+Lyo+ and TFF/DF-sucrose+Lyo+ workflows differed from conventional ultracentrifugation-derived preparations in some recovery metrics: protein-associated recovery was lower, whereas lipid-associated fluorescence and particle counts were not significantly different among workflows. Despite these differences, ultracentrifugation-free preparations retained critical OMV attributes, including vesicular morphology, nanoscale particle size, broadly preserved protein profiles, membrane-protected mCherry-FLAG cargo, and detectable reporter fluorescence. These findings support the feasibility of combining TFF-based preconcentration with subsequent diafiltration in the same TFF system, followed by lyophilization and reconstitution into a coherent research-grade downstream workflow. The observed differences among protein-, lipid-, and particle-based readouts may reflect changes in vesicle-associated material, soluble contaminants, vesicle subpopulations, or workflow-dependent effects on particle detectability rather than generalized OMV loss. Although particle concentrations measured by NTA were numerically lower in the TFF/DF-NaCl+Lyo+ and TFF/DF-sucrose+Lyo+ workflows than in ultracentrifugation-derived preparations, these differences were not statistically significant under the conditions tested. Therefore, these data should be interpreted as evidence that ultracentrifugation-free processing did not produce a statistically demonstrable reduction in particle-associated recovery, while acknowledging that NTA provides an operational estimate of light-scattering particles rather than an absolute count of intact OMVs. In this context, reduced numerical particle counts could reflect true vesicle loss during TFF, diafiltration, lyophilization, reconstitution, or final filtration, but they could also arise from increased aggregation, altered particle dispersion, retention of vesicles within the filtration system, or changes in the proportion of particles falling within the optimal detection range of the instrument. Resolving these possibilities will require deeper compositional and process-recovery analyses, including proteomics, lipidomics, LPS profiling, contaminant assessment, mass-balance recovery studies across each processing step, and orthogonal particle characterization approaches. Importantly, retention of detectable β-lactamase activity, mCherry-associated fluorescence, and proteinase K-protected reporter signal after lyophilization should not be interpreted as definitive evidence of complete vesicle preservation, but rather as evidence supporting compatibility of the workflow with maintenance of multiple OMV-associated structural and functional readouts.

The comparison between NaCl- and sucrose-exchanged ultracentrifugation-free workflows suggests that both diafiltration solutions were compatible with preservation of detectable cargo-associated fluorescence after lyophilization. Although TFF/DF-sucrose+Lyo+ preparations showed a numerically higher mCherry fluorescence signal than TFF/DF-NaCl+Lyo+ preparations, this difference was not statistically significant under the conditions tested. Therefore, the present data do not demonstrate a superior protective effect of sucrose, but they remain consistent with the broader formulation rationale that non-reducing sugars such as sucrose or trehalose can stabilize vesicular and protein-containing systems during drying by limiting membrane destabilization, aggregation, or loss of functional signal (Trenkenschuh et al., 2022; Ahmadian et al., 2024). Because sucrose was evaluated here as a diafiltration exchange solution rather than as part of a systematically optimized lyoprotectant formulation, and because the effect was assessed using a single fluorescent reporter cargo, future work should examine sugar concentration, composition of the diafiltration solution, residual moisture, reconstitution conditions, storage duration, and cargo-specific potency readouts.

These findings have broader implications for OMV-based biotechnology. OMVs are being developed as platforms for vaccination, mucosal delivery, antigen display, and bacterial nanobiotechnology, but their implementation remains constrained by purification reproducibility, product definition, scalability, and storage stability (Muralinath et al., 2011; Fantappie et al., 2014; Gerritzen et al., 2017; Howlader et al., 2018; Haque et al., 2021; Sartorio et al., 2021). The workflow described here contributes to this area by combining non-genetic reduction of flagellin carryover, TFF-based concentration, diafiltration-mediated solution exchange, lyophilization, and post-reconstitution functional assessment in *S.* Typhi. This is particularly relevant for *Salmonella*-derived and engineered OMVs, where contaminant profile, vesicle composition, cargo integrity, and recipient-cell interaction can strongly influence downstream interpretation and application. Importantly, OMV quality should not be interpreted as a universal or application-independent property. Rather, the relative importance of each quality attribute is expected to depend on the intended use of the vesicle preparation. For vaccine-oriented applications, antigen composition, intrinsic immunostimulatory activity, contaminant definition, reactogenicity control, and storage stability may be particularly relevant (Muralinath et al., 2011; Gerritzen et al., 2017; Howlader et al., 2018; Haque et al., 2021; Sartorio et al., 2021). In contrast, for delivery-oriented or engineered OMV platforms, cargo loading, membrane-dependent cargo protection, preservation of reporter or enzymatic activity, vesicle dispersion, and recipient-cell interaction may become particularly important OMV quality attributes for these applications (Fantappie et al., 2014; Alves et al., 2016; Fuentes et al., 2025). From this perspective, the framework used here should be viewed as a fitness-for-purpose approach in which process-performance readouts, particularly apparent recovery, are interpreted together with structural, biochemical, physicochemical, cargo-associated, and functional OMV quality attributes according to the intended downstream application. At the same time, the scope of the study should be defined carefully. The present workflow supports standardized research-grade OMV preparation and provides a practical basis for future bioprocess development, but it does not establish GMP-ready manufacturing, clinical equivalence, or universal transferability to other Gram-negative producers.

Several limitations should be considered. First, although the workflow was evaluated using complementary readouts of structural, biochemical, physicochemical, cargo-associated, and functional OMV quality attributes, comparative proteomic, lipidomic, glycomic, or LPS-focused analyses were not performed. Subtle changes in OMV composition, vesicle subpopulations, surface architecture, or low-abundance cargo therefore cannot be excluded. Second, soluble proteins, protein aggregates, flagellar fragments, medium-derived components, and other non-vesicular material were not exhaustively quantified across workflows, even though isolation procedures are known to influence recovery, purity, and co-isolated contaminants (Klimentová and Stulík, 2015; Reimer et al., 2021; Choi and Lee, 2025). Third, DiO/trypan blue assays should be interpreted as cell-associated and quenching-resistant fluorescence readouts rather than direct evidence of preserved OMV uptake behavior, since membrane dyes may associate nonspecifically and fluorescence quenching does not fully distinguish surface-associated from internalized signal. Fourth, reduced flagellin carryover was associated with increased epithelial cell-associated fluorescence, but causality was not directly demonstrated. Fifth, the selected culture condition and ultracentrifugation-free workflows remain to be tested in other Gram-negative bacteria, engineered OMV producers, bioreactor systems, and larger-scale configurations. Finally, lyophilization was performed as a laboratory-scale preservation procedure, not as a validated manufacturing process; future studies should address formulation optimization, residual moisture, sterility assurance, lot-to-lot reproducibility, potency assays, contaminant profiling, and process scale-up before translational or manufacturing claims are made.

In conclusion, this study shows that S. Typhi OMV nanoparticles are shaped across the entire production workflow, from culture conditions to downstream processing and preservation. The data support an integrated process-development framework in which process-performance readouts are evaluated together with application-dependent OMV quality attributes, including morphological definition, particle-size distribution, biochemical profile, surface charge and clustering, contaminant carryover, membrane-dependent cargo protection, and functional activity. Within this framework, the integrated ultracentrifugation-free workflow provides a practical research-grade basis for more standardized and scale-compatible *S*. Typhi OMV nanoparticle processing. By combining upstream reduction of flagellin-associated contaminant carryover with TFF-based concentration, subsequent diafiltration in the same TFF system, lyophilization, and post-reconstitution functional assessment, this work supports the development of OMVs as process-defined microbial biotechnology platforms, while identifying clear priorities for compositional profiling, formulation development, and process validation.

## Data Availability

The original contributions presented in the study are included in the article/Supplementary material, further inquiries can be directed to the corresponding authors.
