# Supplementary material for: Toward ultracentrifugation-free processing and long-term preservation of outer membrane vesicles by culture optimization, tangential flow filtration, diafiltration, and lyophilization
Source: Front Microbiol. 2026 Aug 31;17:1927950. doi: 10.3389/fmicb.2026.1927950 (PMC13572657; doi:10.3389/fmicb.2026.1927950)
Supplement: Supplementary file 1 [file Supplementary_file_1.docx]

**SUPPLEMENTARY** **MATERIAL**

**Toward Ultracentrifugation-Free Processing and Long-term Preservation of Outer Membrane Vesicles by Culture Optimization, Tangential Flow Filtration, Diafiltration, and Lyophilization**

Andres Silva¹˒²†, Ignacio Fuentes¹˒²†, Jessenia C. Melio¹˒³, Francisco Parra¹˒², Diana Majluf¹˒⁴, Diego Rojas¹˒², Felipe M. Llancalahuen⁵, Maria J. Barros⁶, Katina Schinnerling⁷, Fernando Gil⁸˒⁹*, Iván L. Calderón⁶* and Juan A. Fuentes¹*.

1. Laboratorio de Genética y Patogénesis Bacteriana, Centro de Investigación de Resiliencia a Pandemias, Facultad de Ciencias de la Vida, Universidad Andrés Bello, Santiago, Chile.
2. Doctorado en Biotecnología, Facultad de Ciencias de la Vida, Universidad Andrés Bello, Santiago, Chile.
3. Doctorado en Biociencias Moleculares, Facultad de Ciencias de la Vida, Universidad Andrés Bello, Santiago, Chile.
4. Magíster en Biotecnología y Ciencias de la Vida, Facultad de Ciencias de la Vida, Universidad Andrés Bello, Santiago, Chile.
5. Translational Research Laboratory in Intensive Care Medicine, Departamento de Medicina intensiva, Facultad de Medicina, Pontificia Universidad Católica de Chile, Santiago, Chile.
6. Laboratorio de RNAs Bacterianos, Centro de Investigación de Resiliencia a Pandemias, Facultad de Ciencias de la Vida, Universidad Andrés Bello, Santiago, Chile.
7. Laboratorio de Inmunología Traslacional, Centro de Investigación de Resiliencia a Pandemias, Facultad de Ciencias de la Vida e Instituto de Salud Pública, Universidad Andrés Bello, Santiago, Chile.
8. School of Medicine, Faculty of Medicine, Universidad de los Andes, Santiago, Chile.
9. Microbiota-Host Interactions and Clostridia Research Group, Center for Biomedical Research and Innovation (CIIB), Universidad de los Andes, Santiago, Chile.

† These authors contributed equally to this work and share first authorship

* Corresponding author

**Supplementary figures:**

**Supplementary figure 1. Verification and motility characterization of the Δ*fliC*::FRT mutant in *Salmonella* Typhi. (A)** Molecular confirmation of the *Salmonella* Typhi Δ*fliC*::FRT mutant by PCR using primers external to the *fliC* gene in the WT strain and the Δ*fliC*::FRT mutant. A no-template negative control was included. **(B)** Motility assay in 0.3% semi-solid agar comparing the *Salmonella* Typhi WT strain and the Δ*fliC*::FRT mutant after 24 h of incubation. The mutant showed a marked reduction in the motility halo compared with the wild-type strain. Scale bars: 1 cm. **(C)** Quantification of the motility diameter shown in panel B after 24 h. Data are presented as mean ± SEM from three independent biological replicates (n = 3). Statistical significance was determined using an unpaired two-tailed Student’s *t*-test. *p* < 0.01.

**Supplementary Figure 2.** **Protein and lipid content per outer membrane vesicle (OMV) particle obtained from *S.* Typhi cultures grown at 120 rpm and 180 rpm**. (A) Protein content per OMV particle (µg/particle). (B) Lipid content per OMV particle measured using the FM4-64 probe and expressed as arbitrary fluorescence units per particle (AFU/particle). Data are presented as mean ± SEM from three independent biological replicates. No statistically significant differences were detected between conditions (two-tailed Student’s *t*-test).

**Supplementary Figure 3. Relative protein and lipid content per OMV in TFF and NFF derived preparations.**(A) Protein content normalized to particle number (µg/particle) in OMVs obtained by tangential flow filtration (TFF) or normal flow filtration (NFF). (B) Lipid-associated fluorescence normalized to particle number (AFU/particle) in OMVs obtained under the same conditions. Particle concentration was determined by nanoparticle tracking analysis (NTA), and values are expressed as relative OMV production per particle. This normalization enables comparison of the approximate protein and lipid content per vesicle across extraction methods. Data are presented as mean ± SEM from three independent biological replicates. No statistically significant differences were detected between conditions (two-tailed Student’s *t*-test).

**Supplementary Figure 4. Relative protein and lipid content per OMV across conventional and ultracentrifugation-free preparation workflows.** (A) Protein content normalized to particle number (µg/particle) in OMVs obtained under four preparation conditions: ultracentrifugation without lyophilization (TFF/UC+Lyo−), ultracentrifugation followed by lyophilization (TFF/UC+Lyo+), and ultracentrifugation-free workflows based on TFF with diafiltration using NaCl or sucrose, followed by lyophilization. (B) Lipid-associated fluorescence normalized to particle number (AFU/particle) for the same conditions. Particle concentration was determined by nanoparticle tracking analysis (NTA), and values are expressed as relative protein and lipid content per vesicle. This normalization allows comparison of the approximate abundance of protein- and lipid-associated material per OMV across preparation strategies. Data are presented as mean ± SEM from independent three biological replicates.

**Supplementary Figure 5. Gating strategy (Fig. 3C-D; Fig. 8B-E).** Representative flow cytometry plots showing the sequential gating strategy applied to define the final cell population used for analysis. (A) HT-29 cells were first selected according to their forward scatter area and side scatter area profile (FSC-A vs SSC-A) to exclude debris and events with abnormal size or granularity. (B) Single cells were then identified using FSC-A vs FSC-W to remove cell aggregates and doublets. (C) An additional singlet gate was applied using SSC-A vs SSC-W to further refine the single-cell population. (D) Viable cells were selected by excluding PI-positive non-viable cells. (E) Representative control plot showing the fluorescence distribution in the FITC channel, corresponding to the signal of DiO-labeled OMVs, after application of the previous gates. This control population was used to define the fluorescence threshold for positive events in subsequent analyses.

**Supplementary Tables:**

**Supplementary Table 1. Nanoparticle tracking analysis of *S.* Typhi OMV preparations obtained after TFF- or NFF-based preconcentration followed by ultracentrifugation.** OMVs were prepared from *S.* Typhi cultures grown under the selected low-agitation condition, 120 rpm in 250 mL culture volumes. Clarified and filtered culture supernatants were preconcentrated using either tangential flow filtration (TFF) or normal-flow filtration (NFF) before OMV recovery by ultracentrifugation. The resulting preparations were analyzed by nanoparticle tracking analysis (NTA) to determine particle size distribution and concentration. Mean particle diameter, modal diameter, and particle concentration are reported for each condition. Both TFF- and NFF-derived preparations contained particles within the expected nanometric size range for bacterial OMVs and showed comparable particle concentrations under the tested conditions. Data are presented as mean ± SEM from three independent biological replicates. The TFF condition corresponds to the same low-agitation TFF-derived reference preparation used in Table 2 and is shown here to enable direct comparison with the NFF-derived preparation.

| **OMVs source** | **Revolutions per min (rpm)** | **Ultrafiltration** | **Mean size (nm)** | **Mode (nm)** | **Concentration (particles/mL)** |
| --- | --- | --- | --- | --- | --- |
| *S.* Typhi | 120 rpm | TFF | 140.6 ± 4.6 | 105.3 ± 4.2 | 3.92 × 10^11^ ± 1.93 × 10^10^ |
| *S.* Typhi | 120 rpm | NFF | 117.5 ± 2.2 | 94.8 ± 1.4 | 3.45 × 10^11^ ± 1.69 × 10^10^ |
